# Combined Arctigenin and Curcumin Treatment Induces Redox and Metabolic Stress, AMPK Phosphorylation, and Apoptotic Signaling in Prostate Cancer Cells

**DOI:** 10.3390/nu18182968

**Published:** 2026-09-10

**Authors:** Moon-Kyun Cho, Sang-Han Lee, Hae-Seon Nam, Dongsic Choi, Yoon-Jin Lee

**Affiliations:** 1 Division of Molecular Cancer Research, Soonchunhyang Medical Research Institute, Soonchunhyang University, Cheonan 31511, Republic of Korea; mkcho@schmc.ac.kr (M.-K.C.); namhs@sch.ac.kr (H.-S.N.); dongsicchoi@gmail.com (D.C.); 2 Department of Dermatology, Soonchunhyang University Hospital, Seoul 04401, Republic of Korea; 3 Department of Biochemistry, College of Medicine, Soonchunhyang University, Cheonan 31511, Republic of Korea; m1037624@sch.ac.kr; 4 Department of Tropical Medicine, College of Medicine, Soonchunhyang University, Cheonan 31511, Republic of Korea

**Keywords:** arctigenin, curcumin, prostate cancer, reactive oxygen species, metabolic stress, redox homeostasis, AMPK, apoptosis, three-dimensional spheroids

## Abstract

**Background/Objectives:** Prostate cancer remains a major cause of cancer-related mortality in men. Arctigenin (ATG) and curcumin (CUR) exhibit anticancer activity; however, their combined effects on redox and metabolic stress remain incompletely understood. This study investigated the anticancer effects of combined ATG and CUR treatment in prostate cancer cells. **Methods:** PC-3 prostate cancer cells and normal human prostate epithelial (HPrEC) cells were treated with ATG and CUR alone or in combination. Cell viability, combination index, reactive oxygen species (ROS), GSH/GSSG ratio, ATP levels, AMPK phosphorylation, and apoptosis-related responses were evaluated. The contribution of oxidative stress was examined using N-acetyl-L-cysteine (NAC), and treatment effects were further assessed in three-dimensional (3D) spheroids. **Results:** Combined ATG and CUR treatment produced greater growth-inhibitory and apoptotic responses in PC-3 cells than in HPrEC cells and was synergistic under the tested condition in PC-3 cells. The combination increased ROS, reduced the GSH/GSSG ratio and ATP levels, increased AMPK phosphorylation, and enhanced apoptosis-related responses. NAC attenuated ROS accumulation, partially restored cell viability and ATP levels, and reduced caspase-3/7 activity in PC-3 cells. In 3D PC-3 spheroids, NAC also partially restored viability and ATP levels and attenuated increases in cleaved caspase-3 and cleaved PARP. **Conclusions:** Combined ATG and CUR treatment induces redox and metabolic stress and enhanced apoptotic responses in PC-3 cells, with weaker effects in HPrEC cells. NAC rescue supports a functional contribution of oxidative stress. These findings support an association between ROS-related metabolic stress, AMPK phosphorylation, and apoptotic signaling, while precise causal relationships require further investigation.

## 1. Introduction

Prostate cancer is the second most frequently diagnosed malignancy and one of the leading causes of cancer-related mortality among men worldwide [1,2]. Despite substantial advances in early diagnosis and therapeutic interventions, advanced and metastatic prostate cancer remain major clinical challenges because of disease progression, recurrence, and the eventual development of castration-resistant prostate cancer (CRPC), which is associated with poor prognosis and limited therapeutic options [3,4,5]. Current therapeutic strategies, including androgen deprivation therapy, androgen receptor signaling inhibitors, chemotherapy, and taxane-based regimens, have significantly improved patient survival; however, their long-term clinical efficacy is frequently compromised by acquired therapeutic resistance, tumor heterogeneity, and treatment-associated toxicities [6,7,8,9]. In addition to genetic and epigenetic alterations, accumulating evidence indicates that metabolic reprogramming is a fundamental hallmark of prostate cancer progression, enabling cancer cells to adapt to oxidative stress, nutrient limitation, and fluctuating energy demands within the tumor microenvironment [10,11,12,13]. Beyond supporting rapid proliferation, these metabolic adaptations promote tumor survival, therapeutic resistance, and disease progression by maintaining cellular bioenergetics and redox balance under metabolically stressful conditions. Consequently, therapeutic strategies targeting metabolic vulnerabilities and mitochondrial dysfunction have emerged as promising approaches for suppressing tumor progression, overcoming adaptive resistance, and minimizing systemic toxicity [14,15,16].

**Figure 1:**
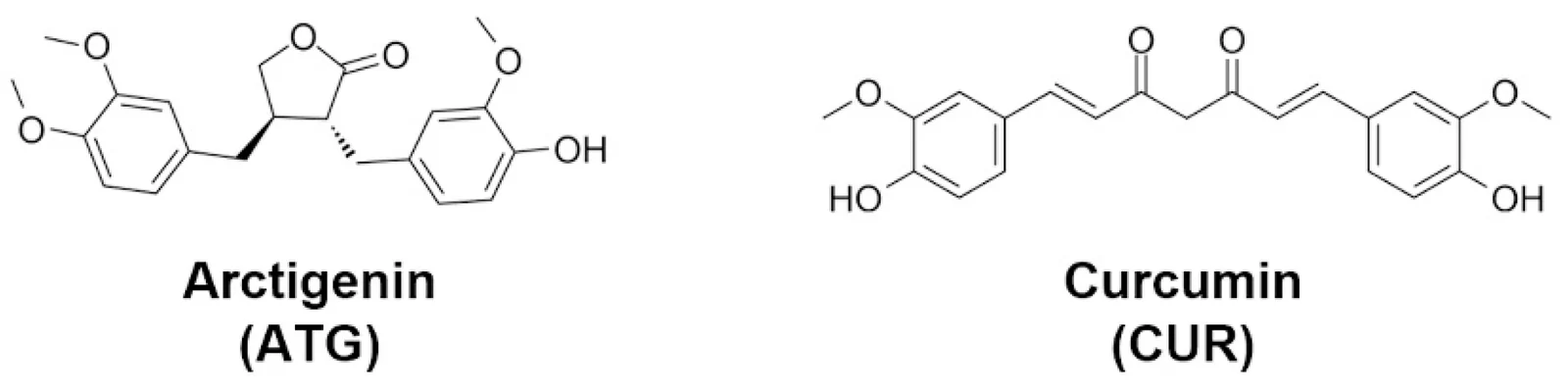
Chemical structures of arctigenin (ATG) and curcumin (CUR).

Naturally derived phytochemicals have emerged as promising candidates for cancer prevention and therapy because of their ability to simultaneously modulate multiple molecular pathways involved in oxidative stress, inflammation, mitochondrial function, cellular metabolism, and programmed cell death while exhibiting relatively low toxicity toward normal tissues [17,18,19,20]. Among these bioactive compounds, arctigenin (ATG), a dibenzylbutyrolactone lignan isolated from Arctium lappa, and curcumin (CUR), a polyphenolic diarylheptanoid derived from Curcuma longa (Figure 1), have attracted considerable attention because of their diverse pharmacological activities, including antioxidant, anti-inflammatory, antiproliferative, and pro-apoptotic effects [21,22,23,24]. Previous studies have demonstrated that ATG suppresses cancer cell growth by regulating mitochondrial function, glycolytic metabolism, AMP-activated protein kinase (AMPK) signaling, and apoptosis, whereas CUR exerts anticancer effects through modulation of multiple oncogenic pathways, including PI3K/Akt, NF-κB, MAPK, and mitochondrial apoptotic signaling [25,26,27,28,29,30]. ATG and CUR were selected for combination treatment because their reported biological activities converge on redox regulation and mitochondrial energy metabolism while involving partially distinct molecular targets. This mechanistic complementarity provided a rationale for examining whether simultaneous exposure could impose greater oxidative and metabolic stress on prostate cancer cells than either compound alone. Although direct evidence regarding combined ATG/CUR treatment remains limited, their overlapping effects on redox homeostasis, cellular metabolism, and mitochondrial apoptotic signaling suggested the potential for an enhanced cooperative anticancer response [31,32].

Accumulating evidence indicates that disruption of redox homeostasis and cellular energy metabolism is a critical determinant of prostate cancer cell fate and has emerged as a promising therapeutic target [33,34,35,36]. Owing to their high metabolic demand and rapid proliferation, prostate cancer cells undergo extensive metabolic reprogramming to adapt to oxidative stress, nutrient deprivation, and fluctuating energy requirements within the tumor microenvironment [37,38,39]. When intracellular antioxidant defenses are overwhelmed, excessive reactive oxygen species (ROS) accumulation results in glutathione (GSH) depletion, ATP depletion, and redox imbalance, ultimately leading to metabolic stress [40,41,42,43]. These metabolic perturbations subsequently activate AMP-activated protein kinase (AMPK), which functions as a central metabolic checkpoint that integrates cellular energy status with survival and stress-response signaling [44,45,46]. However, persistent metabolic stress that exceeds the cellular adaptive capacity induces mitochondrial dysfunction and activates the intrinsic apoptotic pathway, characterized by an increased Bax/Bcl-2 ratio, caspase-3 activation, and PARP cleavage, ultimately leading to irreversible cancer cell death [47,48,49]. Therefore, therapeutic strategies that simultaneously disrupt redox homeostasis and cellular energy metabolism may effectively exploit the metabolic vulnerabilities of prostate cancer cells, providing a strong rationale for the development of metabolism-targeted combination therapies using naturally derived phytochemicals [50].

Despite accumulating evidence demonstrating the individual anticancer activities of ATG and CUR, the effects of their combined treatment on redox homeostasis and cellular energy metabolism in prostate cancer remain insufficiently understood [51,52,53,54]. Moreover, the relationships among oxidative stress, ATP depletion, AMPK activation, and mitochondrial apoptosis following ATG/CUR co-treatment have not been fully defined. We therefore hypothesized that combined ATG/CUR treatment would enhance oxidative and metabolic stress in prostate cancer cells and that ROS generation would contribute functionally to the resulting growth-inhibitory and apoptotic responses. To test this hypothesis, we evaluated cell viability, drug interaction at the selected concentration, intracellular ROS generation, the GSH/GSSG ratio, ATP levels, AMPK phosphorylation, apoptosis-associated signaling, and three-dimensional (3D) spheroid responses in human prostate cancer PC-3 cells. Normal human prostate epithelial (HPrEC) cells were included to assess the relative selectivity of the combined treatment. In addition, N-acetyl-L-cysteine (NAC) rescue experiments were performed in both 2D monolayer and 3D spheroid models to evaluate the functional contribution of oxidative stress to the observed anticancer effects. Collectively, this study examines the relationship between ATG/CUR-induced oxidative and metabolic stress and associated growth-inhibitory and apoptotic responses in prostate cancer cells.

## 2. Materials and Methods

### 2.1. Reagents and Antibodies

Arctigenin (ATG), curcumin (CUR), and N-acetyl-L-cysteine (NAC) were purchased from Sigma-Aldrich (St. Louis, MO, USA). Stock solutions of ATG and CUR were prepared in dimethyl sulfoxide (DMSO) and diluted with the appropriate culture medium immediately before each experiment to obtain the desired working concentrations. The final DMSO concentration did not exceed 0.1% (*v*/*v*) in any experimental group, including the vehicle control, thereby minimizing solvent-related cytotoxicity.

Primary antibodies against phospho-AMPK (Thr172), AMPKα, cleaved caspase-3, caspase-3, cleaved PARP, and PARP were obtained from Cell Signaling Technology (Danvers, MA, USA). The β-actin antibody was purchased from Sigma-Aldrich (St. Louis, MO, USA), and horseradish peroxidase (HRP)-conjugated secondary antibodies were obtained from Cell Signaling Technology. Detailed information regarding the primary antibodies is summarized in Table 1.

### 2.2. Cell Culture

Human prostate cancer PC-3 cells (ATCC, Manassas, VA, USA) and normal human prostate epithelial cells (HPrEC; Lonza, Walkersville, MD, USA) were employed to investigate the selective anticancer activity of ATG and CUR. PC-3 cells were cultured in Dulbecco’s Modified Eagle Medium (DMEM; Welgene, Gyeongsan, Republic of Korea) supplemented with 10% fetal bovine serum (FBS; Gibco, Grand Island, NY, USA), 100 U/mL penicillin, and 100 μg/mL streptomycin. HPrEC cells were maintained in Prostate Epithelial Cell Growth Medium (Lonza, Walkersville, MD, USA) supplemented with the manufacturer’s recommended growth supplements. Both cell lines were maintained at 37 °C in a humidified atmosphere containing 5% CO_2_ and were routinely subcultured before reaching full confluence. Only cells in the exponential growth phase were used throughout the study.

### 2.3. Drug Treatment

For each experiment, PC-3 and HPrEC cells were seeded at assay-specific densities and allowed to adhere overnight before treatment. Fresh working solutions of ATG and CUR were prepared immediately before use by diluting the stock solutions with the corresponding culture medium. Cells were exposed to each compound individually or in combination for 48 h, whereas vehicle-treated controls received an equivalent volume of DMSO (final concentration, 0.1%). Unless otherwise indicated, all subsequent analyses were conducted after 48 h of incubation.

For antioxidant rescue experiments, cells and spheroids were pretreated with NAC (5 mM) for 1.5 h before exposure to ATG (40 μM) and CUR (40 μM). NAC was maintained in the culture medium throughout the subsequent 48 h treatment period.

### 2.4. Evaluation of Antiproliferative Activity

To enhance the reliability of the findings, the antiproliferative activity of ATG and CUR was initially evaluated using the MTT assay and subsequently verified by the trypan blue exclusion assay.

For the MTT assay, HPrEC and PC-3 cells were seeded into 96-well plates at a density of 5 × 10^3^ cells/well and allowed to adhere overnight. Following 48 h exposure to the indicated treatments, MTT solution (5 mg/mL) was added to each well and incubated for 3 h at 37 °C. The culture medium was carefully removed, and the resulting formazan crystals were dissolved in DMSO. Absorbance was recorded at 570 nm using a microplate reader (BioTek Instruments, Winooski, VT, USA). Cell viability was calculated as a percentage of the untreated control.

To further confirm treatment-induced changes in cell survival, viable cells were quantified using the trypan blue exclusion assay. At the end of the treatment period, both adherent and floating cells were harvested, stained with 0.4% trypan blue solution, and manually counted using a hemocytometer under a light microscope (Leica Microsystems, Wetzlar, Germany). Viable cell numbers were expressed relative to the untreated control.

### 2.5. Assessment of Cellular Biomass

Crystal violet staining was performed to qualitatively visualize and quantitatively determine treatment-induced changes in cellular biomass. For qualitative analysis, HPrEC and PC-3 cells were cultured in 6-well plates and exposed to the indicated treatments for 48 h. Cells were subsequently fixed with 4% paraformaldehyde, stained with crystal violet solution, gently rinsed with distilled water to remove excess dye, and photographed using an inverted microscope.

For quantitative analysis, cells cultured under identical experimental conditions in 96-well plates were stained with crystal violet. The retained dye was dissolved in 10% acetic acid, and absorbance was recorded at 590 nm using a BioTek Instruments microplate reader (Winooski, VT, USA). Cellular biomass was expressed relative to the untreated control.

### 2.6. Evaluation of Drug Interaction at the Selected Concentration

The interaction between ATG and CUR at the selected combination concentration was quantitatively evaluated using the Combination Index (CI) method based on the Chou–Talalay principle. A concentration of 40 μM for each compound was selected based on preliminary viability experiments, in which HPrEC cell viability remained relatively well preserved while measurable growth inhibition was observed in PC-3 cells. CI values were calculated from cell viability data using CompuSyn software (version 1.0; ComboSyn Inc., Paramus, NJ, USA). For the predefined combination of ATG (40 μM) and CUR (40 μM), drug interactions were interpreted as synergistic (CI < 1), additive (CI = 1), or antagonistic (CI > 1).

### 2.7. Assessment of Apoptotic Cell Death

Apoptosis induced by the indicated treatments was quantitatively assessed using the Muse™ Annexin V & Dead Cell Assay Kit (Cat. No. MCH100105; Merck KGaA, Darmstadt, Germany). At the end of the 48 h treatment period, both adherent and floating cells were harvested to avoid the loss of detached apoptotic cells. Following washing with phosphate-buffered saline (PBS), the cells were resuspended in assay buffer and stained with the Annexin V & Dead Cell reagent according to the manufacturer’s instructions. Samples were analyzed using a Muse™ Cell Analyzer (Merck KGaA, Darmstadt, Germany), and cell populations were categorized as viable, early apoptotic, late apoptotic, and dead cells.

### 2.8. Measurement of Intracellular Oxidative Stress

Intracellular reactive oxygen species (ROS) production following treatment was determined using the DCFDA/H_2_DCFDA Cellular ROS Assay Kit (Cat. No. ab113851; Abcam, Cambridge, UK). HPrEC and PC-3 cells were seeded into black-walled, clear-bottom 96-well plates at a density of 5 × 10^3^ cells/well and exposed to the indicated treatments for 48 h. The culture medium was subsequently removed, and cells were incubated with DCFDA working solution for 45 min at 37 °C in the dark. Fluorescence intensity was recorded using a microplate reader at excitation and emission wavelengths of 485 and 535 nm, respectively. Intracellular ROS levels were expressed relative to the untreated control.

### 2.9. Evaluation of Cellular Redox Status

Intracellular redox status was evaluated by determining the ratio of reduced glutathione (GSH) to oxidized glutathione (GSSG) using the GSH/GSSG-Glo™ Assay (Cat. No. V6611; Promega, Madison, WI, USA). Following 48 h of incubation, HPrEC and PC-3 cells were processed according to the manufacturer’s instructions to independently quantify total glutathione and GSSG. Luminescence was measured using a microplate luminometer (BioTek Instruments, Winooski, VT, USA), and the intracellular GSH/GSSG ratio was calculated from the corresponding values. The calculated ratio was expressed relative to the untreated control and used as an indicator of intracellular redox status.

### 2.10. Determination of Cellular Energy Status

Intracellular ATP levels were quantified using the CellTiter-Glo^®^ Luminescent Cell Viability Assay (Cat. No. G7570; Promega, Madison, WI, USA) to evaluate treatment-associated changes in cellular energy status. HPrEC and PC-3 cells were seeded into 96-well plates at a density of 5 × 10^3^ cells/well and allowed to adhere overnight. Following exposure to the indicated treatments for 48 h, an equal volume of CellTiter-Glo^®^ reagent was added directly to each well. The plate was mixed on an orbital shaker for 2 min to ensure complete cell lysis and subsequently incubated at room temperature for 10 min to stabilize the luminescent signal. Luminescence was measured using a microplate luminometer, and intracellular ATP levels were expressed relative to the untreated control.

### 2.11. Analysis of AMPK and Apoptosis-Related Proteins

To investigate the molecular alterations associated with the enhanced anticancer effects of ATG/CUR co-treatment, protein expression associated with AMPK signaling and mitochondrial apoptosis was analyzed by Western blotting. Following 48 h of treatment, total cellular proteins were extracted using RIPA lysis buffer supplemented with protease and phosphatase inhibitor cocktails. Protein concentrations were determined using the bicinchoninic acid (BCA) protein assay. Equal amounts of protein (30 μg) were separated by SDS-PAGE on 10–12% polyacrylamide gels and transferred onto polyvinylidene difluoride (PVDF) membranes.

The membranes were blocked with 5% non-fat skim milk for 2 h at room temperature and incubated overnight at 4 °C with the primary antibodies listed in Table 1. Following washing, membranes were incubated with horseradish peroxidase (HRP)-conjugated secondary antibodies for 1 h at room temperature. Immunoreactive bands were visualized using an enhanced chemiluminescence (ECL) detection system, and band intensities were quantified with ImageJ software (version 1.54; National Institutes of Health, Bethesda, MD, USA). Phospho-AMPKα (Thr172) expression was normalized to total AMPKα, whereas cleaved caspase-3, caspase-3, cleaved PARP, and PARP expression levels were normalized to β-actin.

### 2.12. Three-Dimensional Spheroid Culture and Treatment

To evaluate whether the differential effects of ATG/CUR co-treatment were retained under three-dimensional (3D) culture conditions, spheroids were established using HPrEC and PC-3 cells. Briefly, HPrEC and PC-3 cells were seeded at a density of 1 × 10^4^ cells/well into ultra-low attachment (ULA) 96-well plates containing complete culture medium. The plates were centrifuged at 1500 rpm for 15 min to facilitate uniform spheroid formation and subsequently incubated under standard culture conditions until compact spheroids developed. Only uniformly formed spheroids were used for subsequent experiments. Following spheroid formation, the culture medium was replaced with fresh medium containing the indicated treatments, and spheroids were incubated for an additional 48 h. Representative bright-field images were acquired using an inverted phase-contrast microscope to evaluate treatment-induced morphological changes.

For qualitative assessment of spheroid viability, spheroids were stained with fluorescein diacetate (FDA, 5 μg/mL) and propidium iodide (PI, 10 μg/mL) for 5 min at room temperature. Following gentle washing with phosphate-buffered saline (PBS), fluorescence images were acquired using a fluorescence microscope (Leica Microsystems, Wetzlar, Germany). FDA fluorescence identified metabolically active viable cells, whereas PI fluorescence identified membrane-compromised or dead cells. Merged fluorescence images were generated to visualize the spatial distribution of viable and dead cells within individual spheroids.

Quantitative morphological analysis was performed by measuring the equivalent spheroid diameter and projected spheroid area using ImageJ software (National Institutes of Health, Bethesda, MD, USA). Identical analytical parameters were applied to all samples.

To quantitatively assess spheroid viability, the CellVia™ Enhanced Cell Viability Assay Kit (Cat. No. LF-EZ1001A; Young In Frontier Co., Ltd., Seoul, Republic of Korea) was used according to the manufacturer’s instructions. Following treatment, CellVia reagent was added to the spheroids, and the plates were incubated for 1 h under standard culture conditions to allow color development. Absorbance was subsequently measured at 450 nm using a microplate reader. Spheroid viability was expressed relative to the untreated control.

In parallel, spheroid ATP levels were independently quantified using the CellTiter-Glo^®^ Luminescent Cell Viability Assay (Cat. No. G7570; Promega, Madison, WI, USA) according to the procedure described in Section 2.10. Luminescence values were expressed relative to the untreated control and used as an indicator of treatment-associated changes in cellular energy status.

For NAC rescue experiments, HPrEC and PC-3 spheroids were pretreated with NAC (5 mM) for 1.5 h prior to exposure to ATG (40 μM) and CUR (40 μM) for 48 h, with NAC maintained in the culture medium throughout the treatment period. Spheroid viability and ATP levels were evaluated as described above. In addition, apoptosis-related protein expression, including cleaved caspase-3 and cleaved PARP, was analyzed by Western blotting according to the procedures described in Section 2.11.

### 2.13. Statistical Analysis

All experiments were independently performed three times (n = 3 independent experiments), with three technical replicate wells per condition for plate-based assays. Data are presented as the mean ± standard deviation (SD). Statistical analyses were conducted using GraphPad Prism version 10.0 (GraphPad Software, San Diego, CA, USA). Comparisons among multiple groups were performed using one-way analysis of variance (ANOVA) followed by Tukey’s multiple comparison test. Statistical significance was defined as *p* < 0.05 (*), *p* < 0.01 (**), *p* < 0.001 (***), and *p* < 0.0001 (****).

## 3. Results

### 3.1. Effects of ATG and CUR on the Viability of Normal and Prostate Cancer Cells

**Figure 2:**
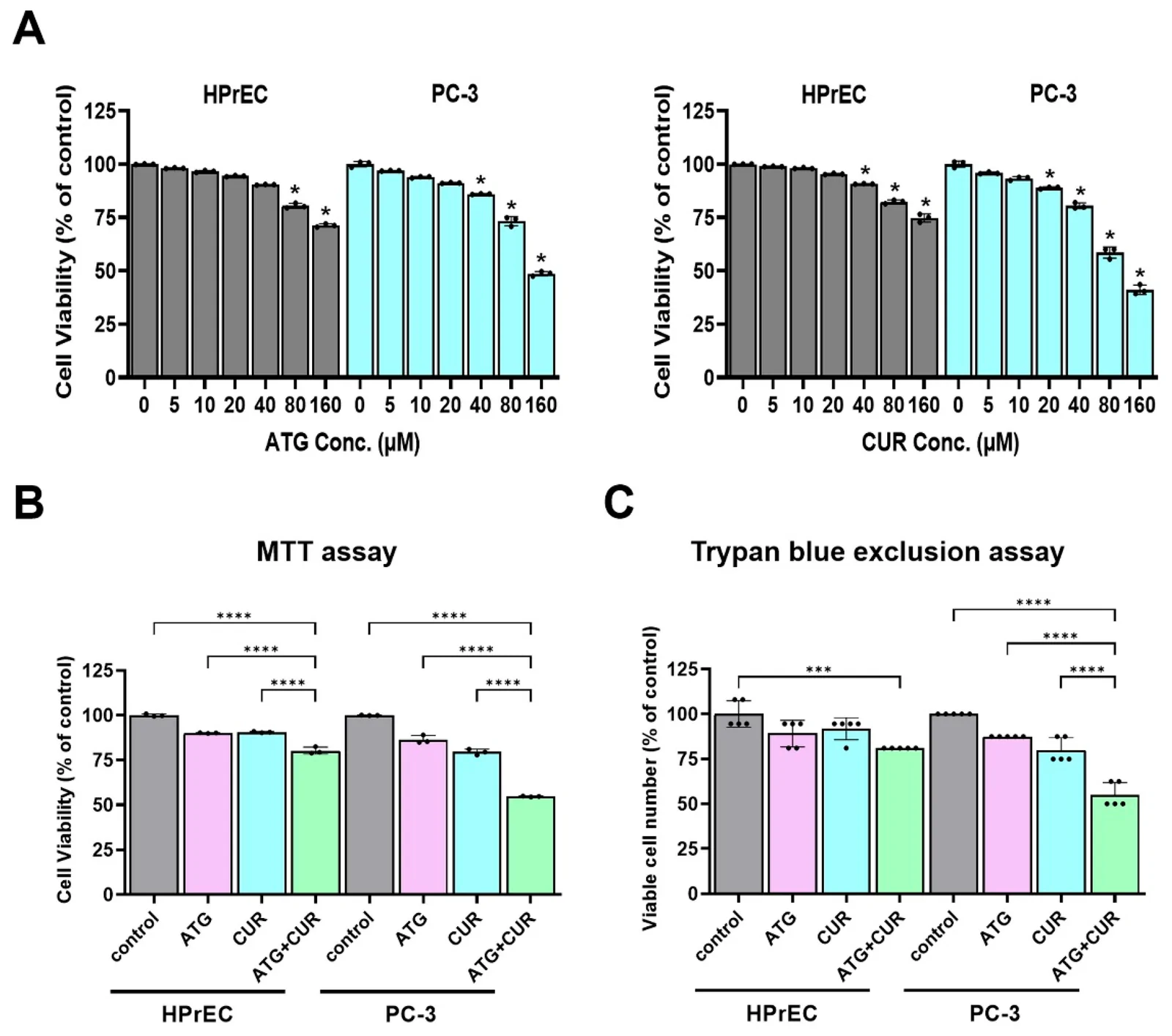
Effects of arctigenin (ATG) and curcumin (CUR) on the viability of HPrEC and PC-3 cells. (**A**) Cell viability of HPrEC and PC-3 cells following treatment with increasing concentrations of ATG or CUR (0–160 μM) for 48 h, as determined by the MTT assay. (**B**) Cell viability following treatment with ATG (40 μM), CUR (40 μM), or their combination (ATG + CUR) for 48 h, as measured by the MTT assay. (**C**) Viable cell number following treatment with ATG (40 μM), CUR (40 μM), or their combination for 48 h, as determined by the trypan blue exclusion assay. Based on these preliminary findings, 40 μM was selected for subsequent experiments because HPrEC cell viability remained approximately 90%, while measurable growth inhibition was observed in PC-3 cells. Data are presented as the mean ± SD from three independent experiments. Statistical significance was analyzed using one-way ANOVA followed by Tukey’s multiple comparison test. * *p* < 0.05, *** *p* < 0.001, and **** *p* < 0.0001.

To determine an appropriate concentration for subsequent mechanistic experiments, we first examined the effects of ATG and CUR on the viability of normal human prostate epithelial (HPrEC) cells and human prostate cancer PC-3 cells. As shown in Figure 2A, both compounds progressively reduced cell viability as the concentration increased in both cell lines. At 40 μM, ATG and CUR maintained greater than 90% viability in HPrEC cells (90.3% and 90.7%, respectively), whereas PC-3 cell viability decreased to 86.0% and 80.5%, respectively. At the highest concentration tested (160 μM), ATG reduced viability to 71.3% in HPrEC cells and 48.7% in PC-3 cells, while CUR decreased viability to 74.8% and 41.1%, respectively. Based on these findings, 40 μM was selected for all subsequent experiments because it preserved normal cell viability while producing measurable growth inhibition in PC-3 cells, thereby providing suitable conditions for evaluating the differential effects of ATG and CUR between PC-3 and HPrEC cells.

Next, we investigated whether combined treatment with ATG and CUR produced greater growth inhibition than either compound alone. Following 48 h of treatment with ATG (40 μM), CUR (40 μM), or their combination, the MTT assay demonstrated that combined treatment reduced PC-3 cell viability to 54.7% of the control, whereas ATG or CUR alone decreased viability to 86.4% and 79.4%, respectively (Figure 2B). By comparison, HPrEC viability remained relatively high following treatment with ATG (90.1%) or CUR (90.6%), with only a modest reduction observed after combined treatment (80.3%).

The trypan blue exclusion assay yielded results consistent with those obtained from the MTT assay (Figure 2C). The viable PC-3 cell population decreased to 87.5%, 80.0%, and 55.0% of the control following treatment with ATG, CUR, and their combination, respectively. Under the same experimental conditions, HPrEC cells maintained viabilities of 89.2%, 91.9%, and 81.1%, respectively. Taken together, these findings indicate that combined treatment with ATG and CUR produced a substantially greater reduction in viability in PC-3 cells than in normal prostate epithelial cells.

### 3.2. Drug Interaction and Antiproliferative Effects of Combined ATG and CUR at the Selected Concentration

To evaluate the interaction between ATG and CUR at the selected concentration, combination index (CI) analysis was performed using ATG (40 μM) and CUR (40 μM). Across three independent experiments, the mean CI value in PC-3 cells was 0.816 ± 0.032, consistent with a synergistic interaction under the tested condition. In HPrEC cells, the mean CI value was 1.088 ± 0.191, which remained close to 1, indicating an interaction close to additivity under the tested condition. Because the CI analysis was performed at a single predefined combination concentration, these findings were interpreted as evidence of drug interaction under the tested condition rather than as generalized pharmacological synergy across a broader dose range.

**Figure 3:**
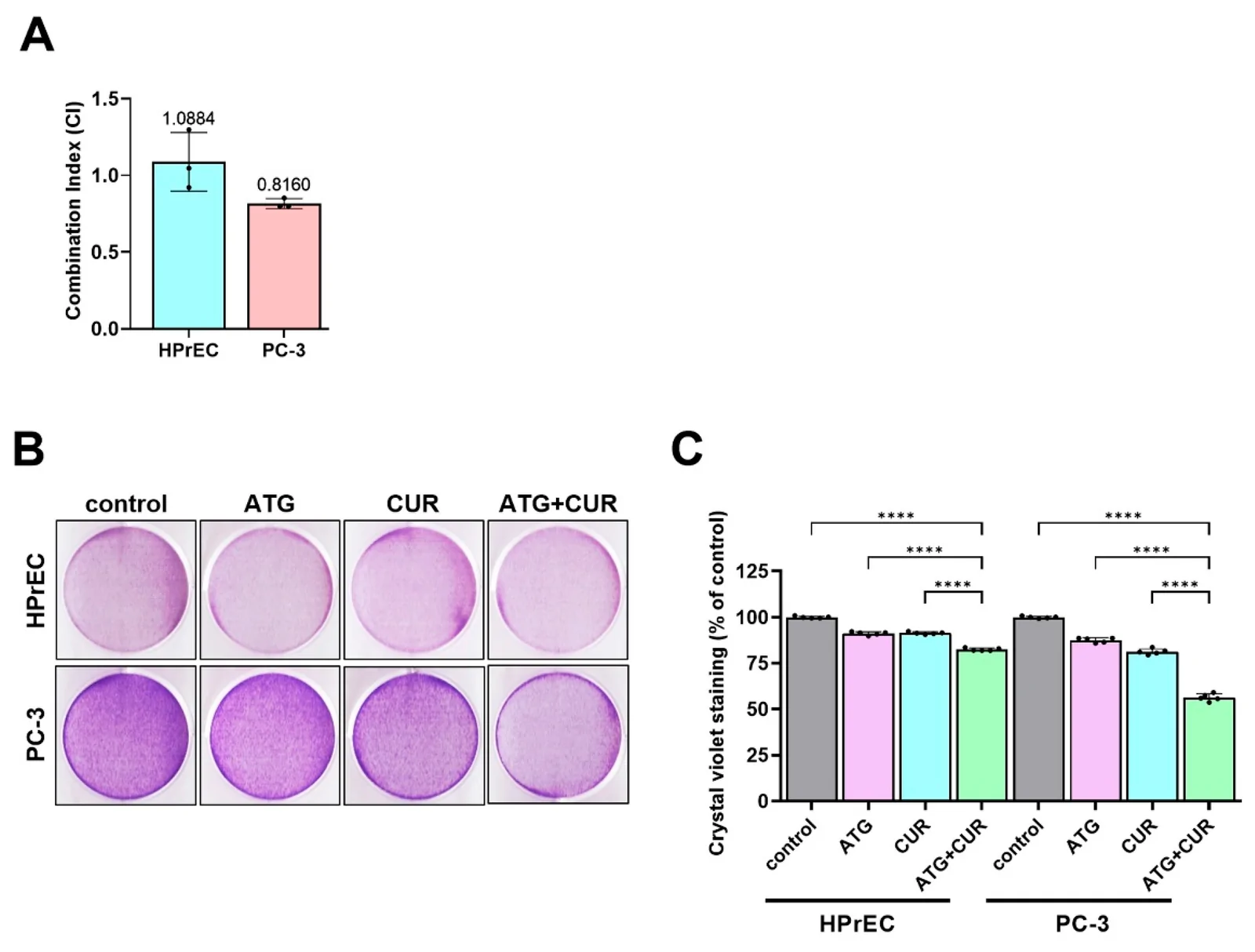
Drug interaction and antiproliferative effects of combined ATG and CUR treatment in HPrEC and PC-3 cells at the selected concentration. (**A**) Combination index (CI) analysis of ATG (40 μM) and CUR (40 μM) co-treatment. Mean CI values were 1.088 ± 0.191 in HPrEC cells and 0.816 ± 0.032 in PC-3 cells, based on three independent experiments. A CI value < 1 indicates synergism, CI = 1 indicates an additive effect, and CI > 1 indicates antagonism. (**B**) Representative crystal violet-stained cultures following treatment with ATG (40 μM), CUR (40 μM), or their combination (ATG + CUR) for 48 h. (**C**) Quantitative analysis of crystal violet staining. Data are presented as the mean ± SD from three independent experiments. Statistical significance was determined using one-way ANOVA followed by Tukey’s multiple comparison test. **** *p* < 0.0001.

The antiproliferative effect of the combined treatment was further evaluated by crystal violet staining (Figure 3B,C). In HPrEC cells, treatment with ATG or CUR alone caused only slight reductions in crystal violet staining, with relative cell densities of 89.9% and 91.0% of the control, respectively. Combined treatment modestly reduced the relative cell density to 81.9%. In contrast, PC-3 cells exhibited substantially greater reductions in crystal violet staining following treatment with ATG (88.6%) or CUR (80.5%) alone. Notably, co-treatment decreased the relative cell density to 57.5% of the control, representing significantly greater growth inhibition than either single treatment (*p* < 0.0001).

Taken together, these findings indicate that, at the selected concentration, combined ATG/CUR treatment produced substantially greater growth inhibition in PC-3 cells than in HPrEC cells, with a CI below 1 observed consistently in PC-3 cells.

### 3.3. Combined Treatment Alters Intracellular Redox Status and Cellular Energy Status in PC-3 Cells

To investigate whether the enhanced antiproliferative effects of ATG and CUR were associated with alterations in intracellular redox status and cellular energy status, intracellular ROS production, the GSH/GSSG ratio, and ATP levels were evaluated following 48 h of treatment.

**Figure 4:**
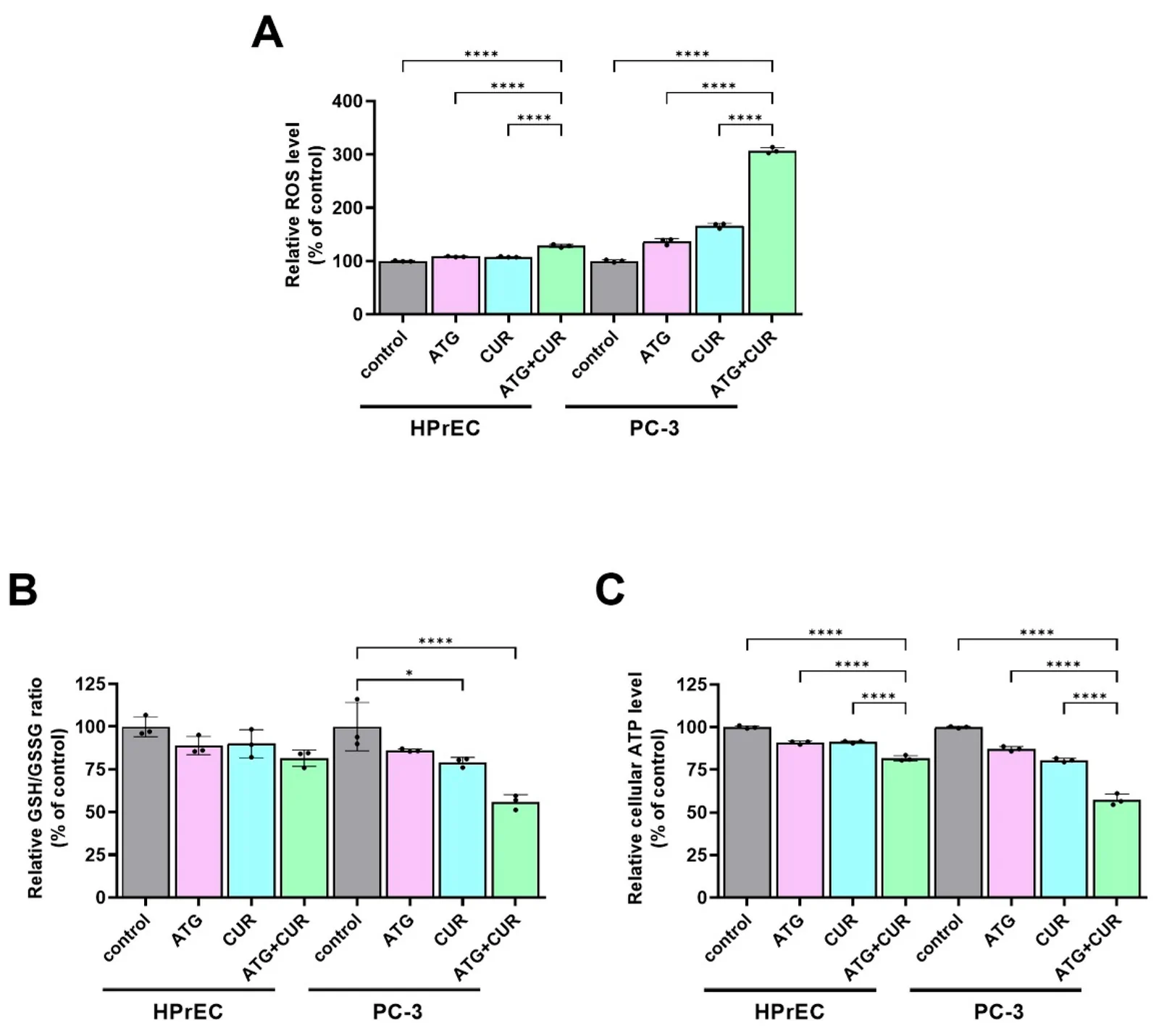
Combined treatment with ATG and CUR alters intracellular redox status and cellular ATP levels in HPrEC and PC-3 cells. (**A**) Intracellular ROS levels in HPrEC and PC-3 cells following treatment with ATG (40 μM), CUR (40 μM), or their combination (ATG + CUR) for 48 h. (**B**) Relative intracellular GSH/GSSG ratio following the indicated treatments. (**C**) Relative intracellular ATP levels quantified using the CellTiter-Glo^®^ Luminescent Cell Viability Assay after 48 h of treatment. Data are presented as the mean ± SD from three independent experiments. Statistical significance was determined using one-way ANOVA followed by Tukey’s multiple comparison test. * *p* < 0.05 and **** *p* < 0.0001.

As shown in Figure 4A, intracellular ROS levels in HPrEC cells showed relatively modest increases following treatment with ATG, CUR, or their combination. In contrast, PC-3 cells exhibited a substantially greater increase in ROS production. ATG alone increased intracellular ROS levels to 117.9% of the control, whereas CUR alone increased ROS levels to 132.3%. Combined treatment further elevated ROS production to 168.1% of the control, representing a significantly greater increase than either single treatment (*p* < 0.0001).

To further evaluate intracellular redox homeostasis, the GSH/GSSG ratio was determined (Figure 4B). Only modest changes were observed in HPrEC cells following either individual or combined treatment. In contrast, combined treatment markedly decreased the GSH/GSSG ratio in PC-3 cells to approximately 56% of the control, whereas ATG and CUR alone reduced the ratio to approximately 86% and 79%, respectively. These findings indicate that combined treatment produced a substantially greater disruption of intracellular redox balance in PC-3 cells than either single treatment.

To further assess treatment-associated changes in cellular energy status, intracellular ATP levels were subsequently measured (Figure 4C). ATP levels in PC-3 cells decreased to 85.9% and 80.6% of the control following treatment with ATG and CUR alone, respectively, and were further reduced to 61.1% by the combined treatment (*p* < 0.0001). By comparison, ATP levels in HPrEC cells remained above 80% under all treatment conditions.

Taken together, these findings indicate that combined ATG/CUR treatment is associated with pronounced redox imbalance and reduced cellular ATP levels in PC-3 cells, with substantially smaller changes observed in HPrEC cells.

### 3.4. Combined Treatment with ATG and CUR Enhances Apoptosis in PC-3 Cells

To determine whether the growth-inhibitory effects of ATG and CUR were associated with apoptotic cell death, apoptosis was evaluated by Annexin V/PI flow cytometry following 48 h of treatment.

**Figure 5:**
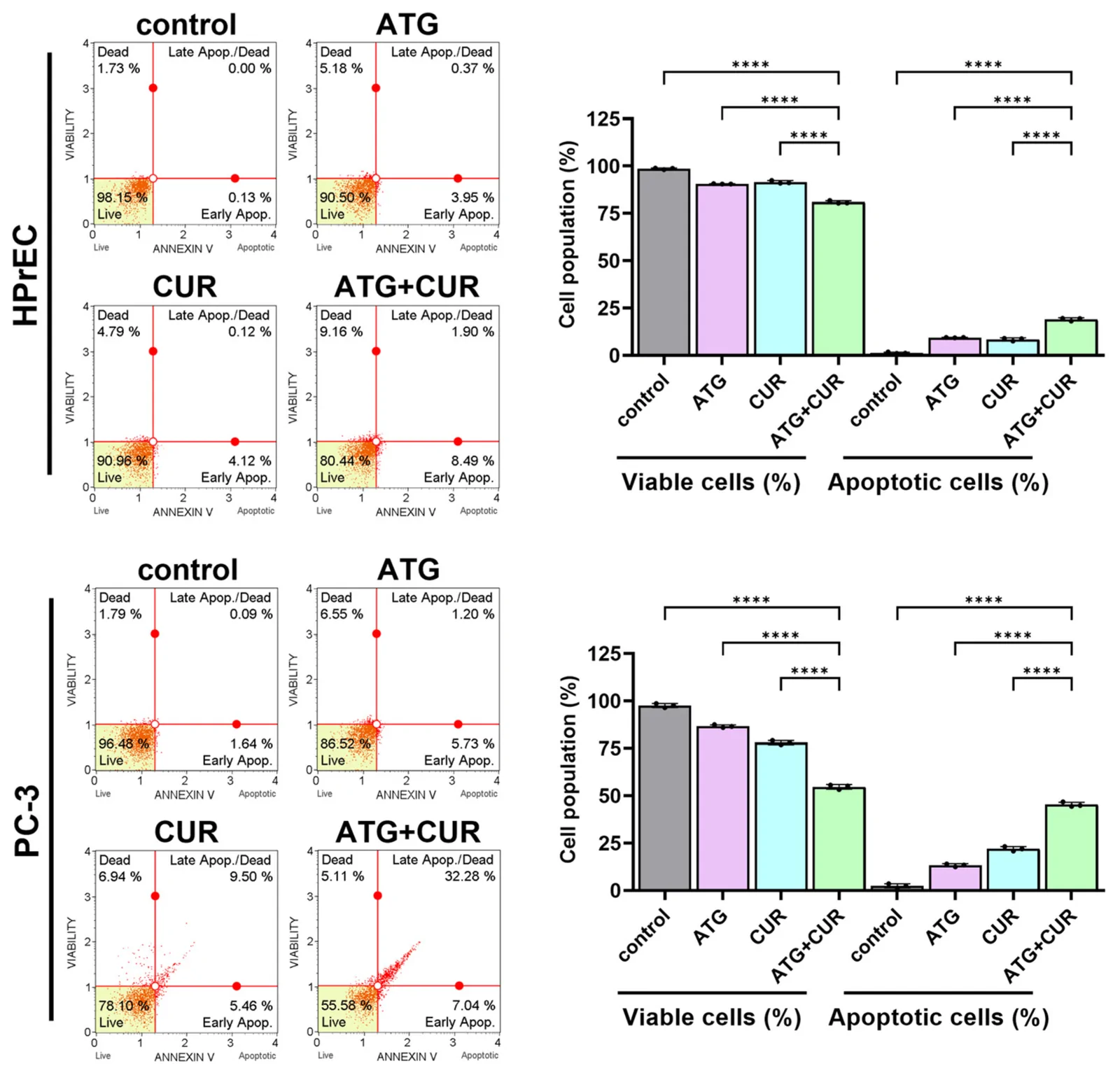
Effects of combined ATG and CUR treatment on apoptosis in HPrEC and PC-3 cells. Representative Annexin V/PI flow cytometry dot plots and quantitative analysis of viable cells and total apoptotic cells (early + late apoptosis) following treatment with ATG (40 μM), CUR (40 μM), or their combination (ATG + CUR) for 48 h. Data are presented as the mean ± SD from three independent experiments. Statistical significance was determined using one-way ANOVA followed by Tukey’s multiple comparison test. **** *p* < 0.0001.

As shown in Figure 5, treatment with ATG or CUR alone increased the proportion of apoptotic cells in both HPrEC and PC-3 cells. However, combined treatment produced a substantially greater apoptotic response in PC-3 cells. In HPrEC cells, the percentage of total apoptotic cells increased from 1.37% in the control group to 9.42% following ATG treatment and 8.48% following CUR treatment. Combined treatment further increased total apoptosis to 19.05%. Despite this increase, the majority of HPrEC cells remained viable under all treatment conditions.

In contrast, apoptosis was markedly enhanced in PC-3 cells. The proportion of total apoptotic cells increased from 2.44% in the untreated control to 13.34% and 21.96% following treatment with ATG and CUR alone, respectively, and was further elevated to 45.35% following combined treatment, representing a significantly greater induction than either single treatment (*p* < 0.0001). Consistent with these findings, the proportion of viable PC-3 cells decreased from 97.56% in the control group to 86.66%, 78.04%, and 54.65% following treatment with ATG, CUR, and their combination, respectively.

Taken together, these findings indicate that combined ATG/CUR treatment produces a substantially greater apoptotic response in PC-3 cells than in HPrEC cells, consistent with the greater growth-inhibitory effect observed in PC-3 cells.

### 3.5. Combined Treatment with ATG and CUR Is Associated with Increased AMPK Phosphorylation and Apoptosis-Related Changes in PC-3 Cells

To further characterize the molecular changes associated with the enhanced apoptotic response induced by combined ATG and CUR treatment, AMPK phosphorylation and the expression of apoptosis-related proteins were examined by Western blot analysis, and caspase-3/7 activity was subsequently evaluated.

**Figure 6:**
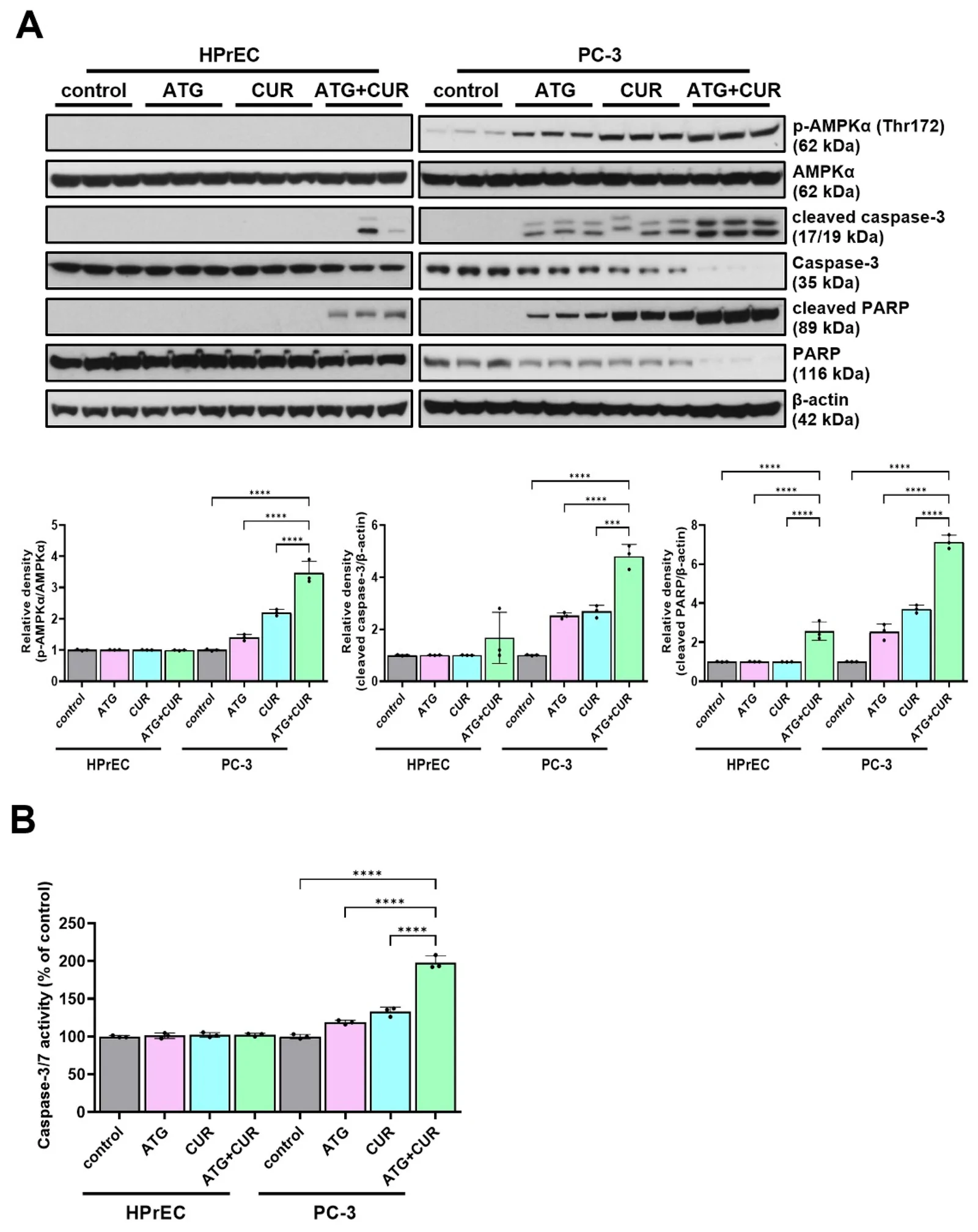
Effects of combined ATG and CUR treatment on AMPK phosphorylation and apoptosis-related markers in HPrEC and PC-3 cells. (**A**) Representative Western blot images and quantitative analysis of phosphorylated AMPKα (Thr172), cleaved caspase-3, and cleaved PARP in HPrEC and PC-3 cells following treatment with ATG (40 μM), CUR (40 μM), or their combination (ATG + CUR) for 48 h. Protein expression levels were normalized to total AMPKα, total caspase-3, total PARP, and β-actin, respectively. (**B**) Relative caspase-3/7 activity following the indicated treatments. Data are presented as the mean ± SD from three independent experiments. Statistical significance was determined using one-way ANOVA followed by Tukey’s multiple comparison test. *** *p* < 0.001 and **** *p* < 0.0001.

As shown in Figure 6A, treatment with ATG or CUR alone moderately increased AMPKα phosphorylation (Thr172) in PC-3 cells, whereas combined treatment produced a markedly greater increase. Quantitative analysis demonstrated that phosphorylated AMPKα expression reached approximately 3.5-fold of the control following combined treatment. In contrast, AMPKα phosphorylation remained essentially unchanged in HPrEC cells under all treatment conditions.

The apoptosis-associated markers cleaved caspase-3 and cleaved PARP also progressively increased following treatment with ATG or CUR alone and were further elevated by combined treatment in PC-3 cells. Combined treatment increased cleaved caspase-3 and cleaved PARP expression to approximately 4.8-fold and 7.1-fold of the control, respectively, whereas only minor changes were observed in HPrEC cells.

To determine whether these molecular changes were accompanied by functional activation of apoptosis, caspase-3/7 activity was subsequently measured (Figure 6B). Consistent with the Western blot findings, caspase-3/7 activity remained essentially unchanged in HPrEC cells but increased from approximately 100% in untreated PC-3 cells to 119.2% and 133.0% following treatment with ATG and CUR alone, respectively. Combined treatment further increased caspase-3/7 activity to approximately 197.9%, representing a significantly greater increase than either single treatment (*p* < 0.0001).

Taken together, these findings show that ATG/CUR co-treatment is associated with increased AMPK phosphorylation and enhanced apoptotic signaling in PC-3 cells, accompanied by increased caspase-3/7 activity. These observations support an association between increased AMPK phosphorylation and the apoptotic response but do not, by themselves, establish a direct causal relationship between these events.

### 3.6. Combined ATG and CUR Treatment Produces Greater Growth-Inhibitory Effects in PC-3 Spheroids than in HPrEC Spheroids

To determine whether the differential effects of combined ATG and CUR treatment observed in two-dimensional (2D) monolayer cultures were also evident in a three-dimensional (3D) context, spheroids were established using HPrEC and PC-3 cells.

**Figure 7:**
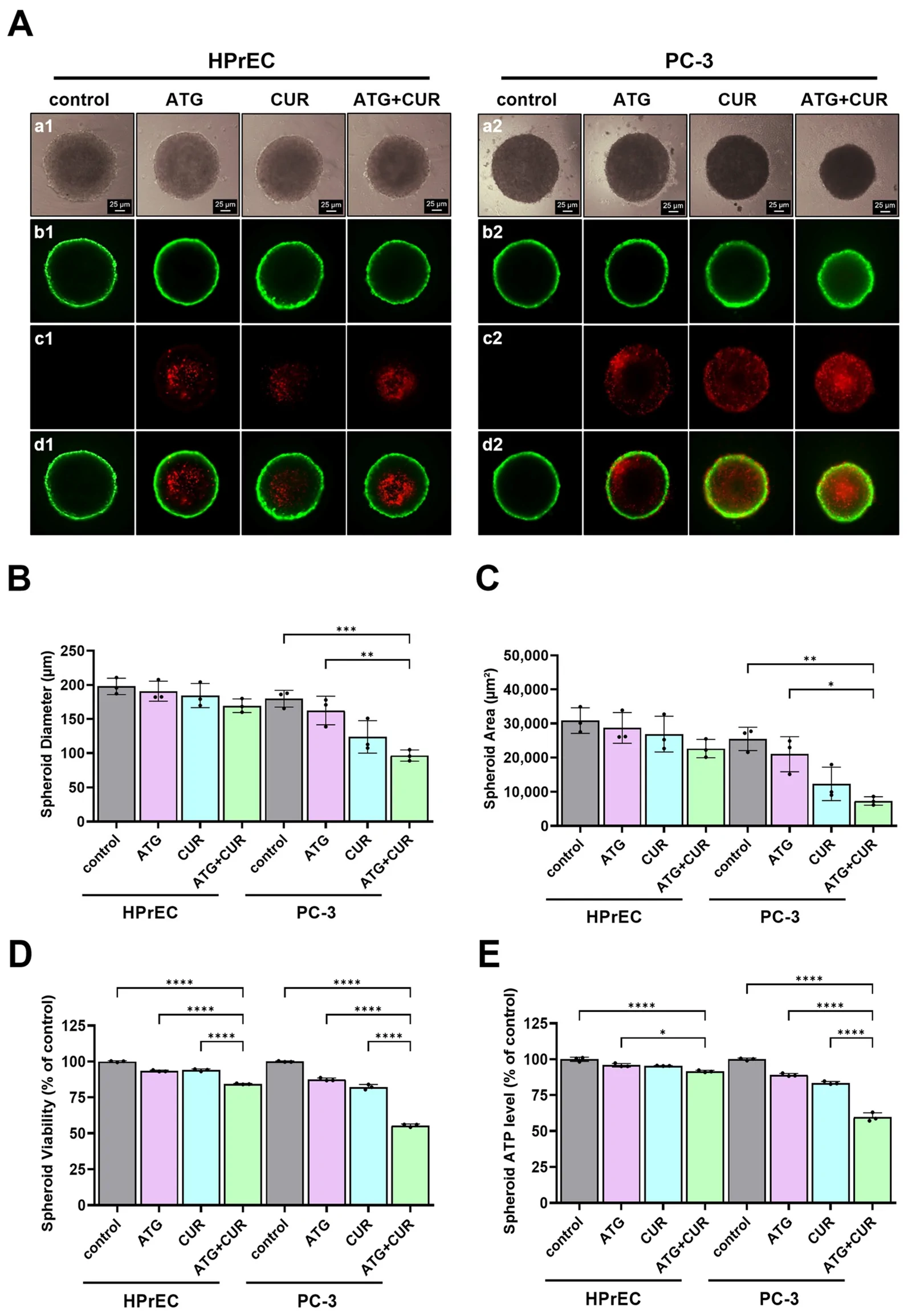
Effects of combined ATG and CUR treatment on HPrEC and PC-3 spheroids. (**A**) Representative bright-field and fluorescence images of HPrEC and PC-3 spheroids following treatment with ATG (40 μM), CUR (40 μM), or their combination (ATG + CUR) for 48 h. Bright-field images (**a1**,**a2**), FDA staining of viable cells (**b1**,**b2**), propidium iodide (PI) staining of dead cells (**c1**,**c2**), and merged fluorescence images (**d1**,**d2**) are shown, where 1 and 2 indicate HPrEC and PC-3 spheroids, respectively. Scale bar = 25 μm. (**B**) Quantitative analysis of spheroid diameter. (**C**) Quantitative analysis of projected spheroid area. (**D**) Relative spheroid viability determined using the CellVia™ Enhanced Cell Viability Assay Kit. (**E**) Relative spheroid ATP levels quantified using the CellTiter-Glo^®^ Luminescent Cell Viability Assay. Data are presented as the mean ± SD from three independent experiments. Statistical significance was determined using one-way ANOVA followed by Tukey’s multiple comparison test. * *p* < 0.05, ** *p* < 0.01, *** *p* < 0.001, and **** *p* < 0.0001.

As shown in Figure 7A, combined treatment induced only minor morphological changes in HPrEC spheroids. In contrast, PC-3 spheroids exhibited marked shrinkage accompanied by a pronounced increase in propidium iodide (PI)-positive staining, consistent with a greater loss of viability within the spheroids.

These morphological observations were supported by quantitative analysis of spheroid size (Figure 7B,C). Only modest reductions in spheroid diameter and projected area were observed in HPrEC spheroids, whereas both parameters were significantly decreased in PC-3 spheroids following combined treatment. Specifically, the mean spheroid diameter decreased from 177.98 μm in the control group to 96.45 μm after combined treatment. Likewise, the projected spheroid area decreased from 25,481 μm^2^ to 7341 μm^2^, representing substantially greater reductions than those observed following either single treatment.

The marked structural alterations in PC-3 spheroids were accompanied by a significant reduction in spheroid viability (Figure 7D). Combined treatment reduced PC-3 spheroid viability to 55.5% of the control, whereas ATG or CUR alone reduced viability to 87.5% and 82.2%, respectively. By comparison, HPrEC spheroids retained 84.4% viability following combined treatment.

To further assess treatment-associated changes in cellular energy status, intracellular ATP levels were evaluated (Figure 7E). ATP levels in HPrEC spheroids remained largely preserved, exceeding 90% of the control under all treatment conditions. In contrast, ATP levels in PC-3 spheroids progressively declined following treatment with ATG (89.1%) or CUR (83.6%) alone and were further reduced to 59.7% of the control following combined treatment.

Taken together, these findings show that the greater growth-inhibitory effect of ATG/CUR co-treatment on PC-3 cells relative to HPrEC cells was also observed in the 3D spheroid model, as reflected by changes in spheroid morphology, viability, and ATP levels.

### 3.7. Combined Treatment with ATG and CUR Is Associated with Increased AMPK Phosphorylation and Apoptosis-Related Changes in Three-Dimensional Spheroids

**Figure 8:**
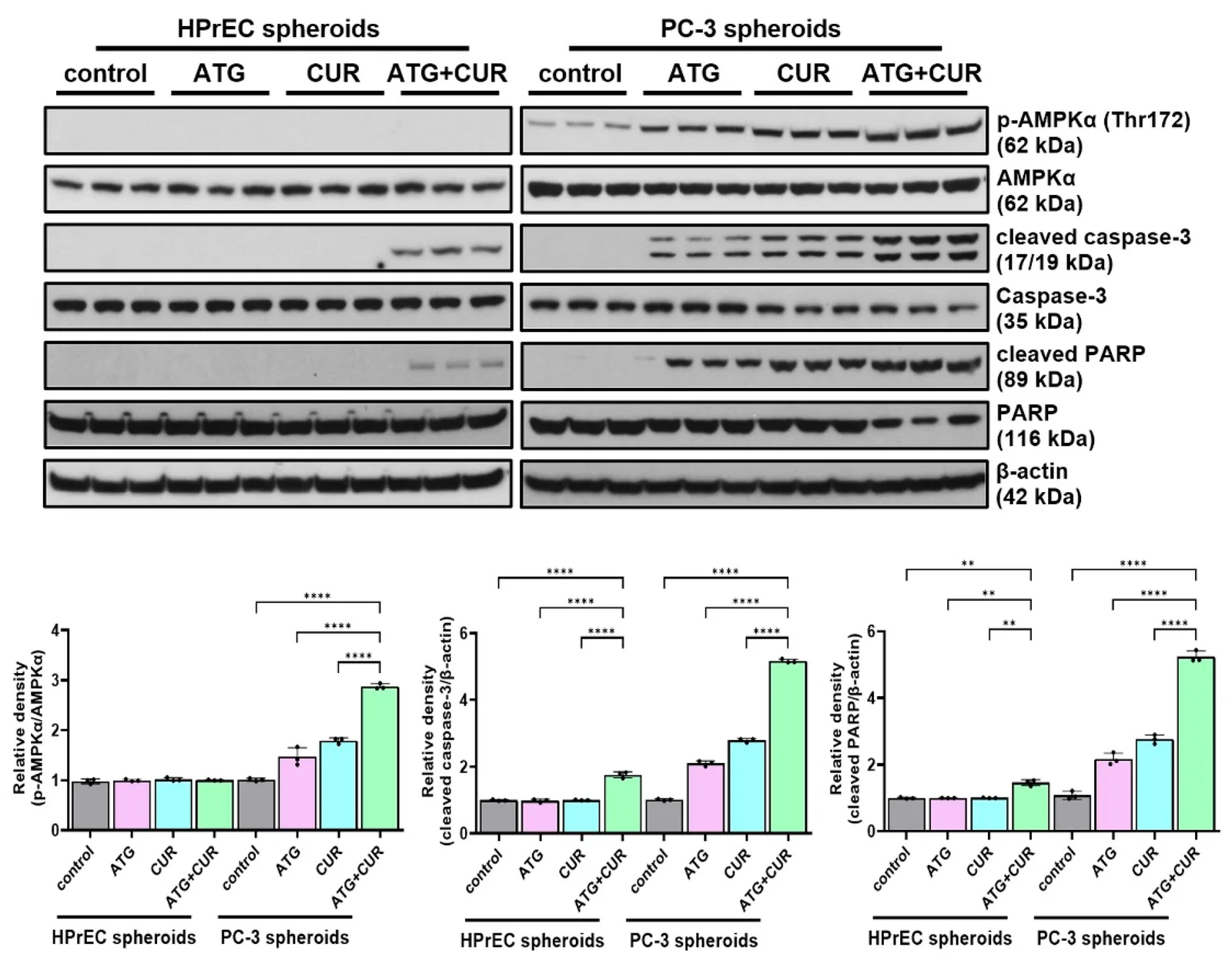
Effects of combined ATG and CUR treatment on AMPK phosphorylation and apoptosis-related markers in HPrEC and PC-3 spheroids. Representative Western blot images and quantitative analysis of phosphorylated AMPKα (Thr172), cleaved caspase-3, and cleaved PARP in HPrEC and PC-3 spheroids following treatment with ATG (40 μM), CUR (40 μM), or their combination (ATG + CUR) for 48 h. Protein expression levels were normalized to total AMPKα, total caspase-3, total PARP, and β-actin, respectively. Data are presented as the mean ± SD from three independent experiments. Statistical significance was determined using one-way ANOVA followed by Tukey’s multiple comparison test. ** *p* < 0.01 and **** *p* < 0.0001.

To determine whether the molecular changes observed in two-dimensional (2D) monolayer cultures were also evident under three-dimensional (3D) culture conditions, AMPK phosphorylation and the expression of apoptosis-related proteins were examined by Western blot analysis in HPrEC and PC-3 spheroids. As shown in Figure 8, AMPKα phosphorylation (Thr172) remained essentially unchanged in HPrEC spheroids following treatment with ATG, CUR, or their combination. In contrast, phosphorylated AMPKα levels progressively increased in PC-3 spheroids following treatment with ATG or CUR alone and were further elevated by combined treatment. Quantitative analysis demonstrated an approximately 2.9-fold increase in phosphorylated AMPKα levels compared with the untreated control.

The apoptosis-associated markers cleaved caspase-3 and cleaved PARP were also increased in PC-3 spheroids following treatment with ATG or CUR alone and were further elevated by combined treatment. Combined treatment increased the levels of cleaved caspase-3 and cleaved PARP to approximately 5.2-fold of the control in PC-3 spheroids, whereas only modest changes were observed in HPrEC spheroids.

Overall, these results show that the increases in AMPK phosphorylation and apoptosis-related protein expression observed in 2D PC-3 cultures were also detected in the 3D spheroid model. These findings support an association between combined ATG and CUR treatment, increased AMPK phosphorylation, and enhanced apoptotic signaling in PC-3 spheroids without establishing a direct causal relationship among these events.

### 3.8. NAC Attenuates ATG/CUR-Induced Changes in Cell Viability, ROS, ATP, and Apoptotic Activity in Two-Dimensional Cultures

To evaluate the functional contribution of oxidative stress to the effects of combined ATG and CUR treatment, HPrEC and PC-3 cells were pretreated with NAC (5 mM) for 1.5 h before exposure to ATG (40 μM) and CUR (40 μM) for 48 h.

**Figure 9:**
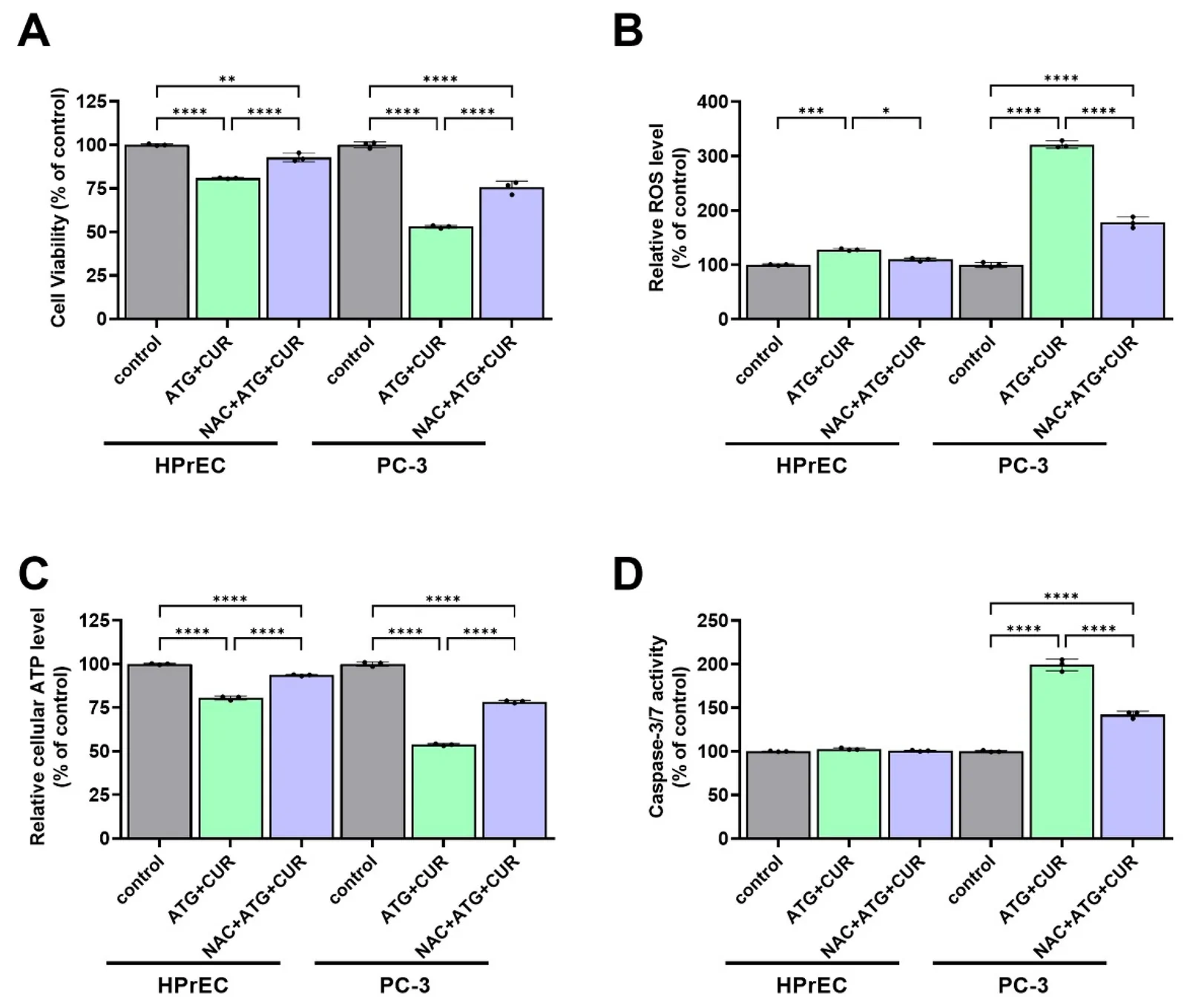
Effects of N-acetyl-L-cysteine (NAC) pretreatment on changes induced by combined ATG and CUR treatment in HPrEC and PC-3 cells. HPrEC and PC-3 cells were pretreated with NAC (5 mM) for 1.5 h before exposure to ATG (40 μM) and CUR (40 μM) for 48 h in the continued presence of NAC. (**A**) Cell viability determined by the MTT assay. (**B**) Relative intracellular reactive oxygen species (ROS) levels. (**C**) Relative cellular ATP levels. (**D**) Relative caspase-3/7 activity. Data are presented as the mean ± SD from three independent experiments. Statistical significance was determined using one-way ANOVA followed by Tukey’s multiple comparison test. * *p* < 0.05, ** *p* < 0.01, *** *p* < 0.001, and **** *p* < 0.0001.

As shown in Figure 9A, combined ATG and CUR treatment reduced HPrEC cell viability to 80.9% of the control, whereas NAC pretreatment partially restored viability to 92.7%. A more pronounced effect was observed in PC-3 cells, in which viability was reduced to 52.9% following combined treatment and was significantly restored to 75.5% by NAC pretreatment (*p* < 0.0001).

NAC also markedly attenuated the increase in intracellular ROS induced by combined ATG and CUR treatment (Figure 9B). In HPrEC cells, relative ROS levels increased to 127.8% following combined treatment and decreased to 109.6% following NAC pretreatment. In PC-3 cells, combined treatment markedly increased ROS levels to 321.1% of the control, whereas NAC pretreatment significantly reduced this increase to 177.6% (*p* < 0.0001).

Changes in cellular ATP levels showed a similar rescue pattern (Figure 9C). Combined treatment reduced ATP levels to 80.5% and 53.8% of the control in HPrEC and PC-3 cells, respectively. NAC pretreatment partially restored ATP levels to 93.6% in HPrEC cells and 78.4% in PC-3 cells (*p* < 0.0001 versus combined treatment in PC-3 cells).

To determine whether attenuation of oxidative stress was accompanied by reduced apoptotic activity, caspase-3/7 activity was subsequently assessed (Figure 9D). In HPrEC cells, caspase-3/7 activity remained close to control levels following either combined treatment (102.6%) or NAC pretreatment (100.7%). In contrast, combined ATG and CUR treatment increased caspase-3/7 activity to 199.1% of the control in PC-3 cells, whereas NAC pretreatment significantly reduced this activity to 142.1% (*p* < 0.0001).

Taken together, these findings show that NAC attenuated ROS accumulation, partially restored cell viability and ATP levels, and reduced caspase-3/7 activity in PC-3 cells. These rescue effects provide functional evidence that oxidative stress contributes, at least in part, to the viability-reducing and apoptotic responses associated with combined ATG and CUR treatment.

### 3.9. NAC Attenuates ATG/CUR-Induced Growth Inhibition and Apoptotic Signaling in Three-Dimensional Spheroids

**Figure 10:**
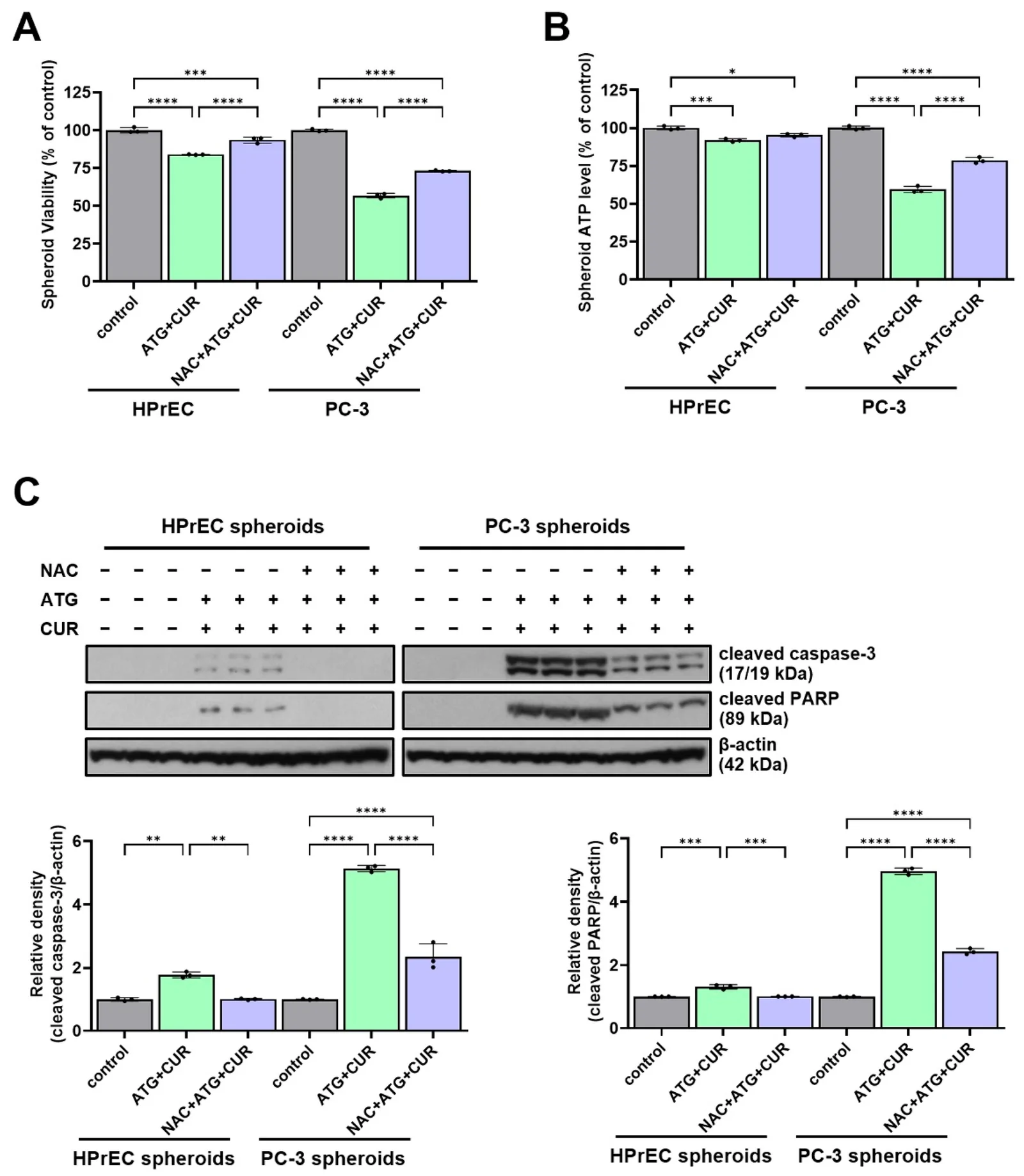
NAC attenuates ATG/CUR-induced growth inhibition, ATP depletion, and apoptotic signaling in HPrEC and PC-3 spheroids. HPrEC and PC-3 spheroids were pretreated with NAC (5 mM) for 1.5 h prior to treatment with ATG (40 μM) and CUR (40 μM) for 48 h. (**A**) Spheroid viability. (**B**) Spheroid ATP levels. (**C**) Representative Western blots and densitometric analysis of cleaved caspase-3 and cleaved PARP in HPrEC and PC-3 spheroids. Protein expression levels were normalized to β-actin. Data are presented as the mean ± SD from three independent experiments. Statistical significance was determined using one-way ANOVA followed by Tukey’s multiple comparison test. * *p* < 0.05, ** *p* < 0.01, *** *p* < 0.001, and **** *p* < 0.0001.

To further determine whether ROS is involved in the effects of combined ATG and CUR treatment in a 3D culture system, HPrEC and PC-3 spheroids were pretreated with NAC (5 mM) for 1.5 h prior to exposure to ATG (40 μM) and CUR (40 μM) for 48 h. As shown in Figure 10A, combined ATG and CUR treatment reduced spheroid viability to 83.9% of the control in HPrEC spheroids and to 56.8% in PC-3 spheroids. NAC pretreatment partially restored spheroid viability to 93.5% in HPrEC spheroids and significantly increased viability to 73.1% in PC-3 spheroids.

A similar pattern was observed for ATP levels (Figure 10B). Combined ATG and CUR treatment reduced ATP levels to 91.8% of the control in HPrEC spheroids and to 59.4% in PC-3 spheroids. NAC pretreatment modestly restored ATP levels to 95.2% in HPrEC spheroids, whereas ATP levels in PC-3 spheroids were significantly restored to 78.6%.

To further examine whether NAC attenuated ATG/CUR-induced apoptotic signaling in 3D spheroids, the levels of cleaved caspase-3 and cleaved PARP were analyzed by Western blotting (Figure 10C). In HPrEC spheroids, combined ATG and CUR treatment caused only a modest increase in cleaved caspase-3 and cleaved PARP, whereas NAC pretreatment reduced both markers to near-control levels. In contrast, PC-3 spheroids showed a marked increase in cleaved caspase-3 and cleaved PARP following combined treatment, and these increases were significantly attenuated by NAC pretreatment. Densitometric analysis confirmed that the combination increased cleaved caspase-3 and cleaved PARP expression to 5.15-fold and 4.97-fold of the control, respectively, in PC-3 spheroids, whereas NAC pretreatment reduced these values to 2.35-fold and 2.43-fold, respectively.

Collectively, these findings indicate that NAC partially attenuated ATG/CUR-induced reductions in spheroid viability and ATP levels, as well as apoptotic signaling, particularly in PC-3 spheroids, supporting a functional contribution of ROS to the effects of the ATG/CUR combination in the 3D spheroid model.

## 4. Discussion

### 4.1. Principal Findings

The present study demonstrates that combined treatment with arctigenin (ATG) and curcumin (CUR) produces substantially greater growth-inhibitory and apoptotic responses in human prostate cancer PC-3 cells than in normal human prostate epithelial (HPrEC) cells under the tested experimental conditions. These findings support the growing concept that simultaneously targeting multiple metabolic vulnerabilities represents an effective therapeutic strategy for selectively eliminating malignant cells while minimizing damage to normal tissues [6,7,8,9,10,11,12,13,14,15,16,31,32].

Rather than acting through a single molecular target, ATG/CUR co-treatment simultaneously disrupted multiple interconnected processes involved in intracellular redox regulation, cellular energy metabolism, and mitochondrial apoptotic signaling. Specifically, combined treatment promoted excessive reactive oxygen species (ROS) accumulation, depleted intracellular glutathione (GSH), reduced ATP production, and activated AMP-activated protein kinase (AMPK). These coordinated molecular alterations were accompanied by an increased Bax/Bcl-2 ratio, activation of caspase-3, and cleavage of PARP, consistent with activation of the intrinsic mitochondrial apoptotic pathway [31,32,33,34,35,36,37,38,39,40,41,42,43,44,45,46,47,48,49,55,56,57,58,59].

Importantly, pretreatment with the ROS scavenger N-acetyl-L-cysteine (NAC) partially reversed the ATG/CUR-induced reductions in cell viability and intracellular ATP levels, providing functional evidence that oxidative stress contributes to the anticancer effects of the combination. The partial rather than complete rescue by NAC further suggests that ROS-dependent mechanisms make an important contribution to ATG/CUR-induced cytotoxicity, while additional ROS-independent mechanisms may also participate in the overall response.

The protective effects of NAC were also observed in the three-dimensional (3D) spheroid model, strengthening the physiological relevance of the findings obtained in conventional monolayer cultures. Combined ATG and CUR treatment markedly reduced spheroid viability and ATP levels in PC-3 spheroids, whereas NAC pretreatment significantly attenuated these effects. In contrast, the corresponding changes were substantially less pronounced in HPrEC spheroids, further supporting the preferential susceptibility of prostate cancer cells to the metabolic and oxidative stress induced by the combination [51,52,53,54,60].

Consistent with these functional findings, Western blot analysis of 3D spheroids demonstrated marked increases in cleaved caspase-3 and cleaved PARP following combined ATG and CUR treatment in PC-3 spheroids. NAC pretreatment significantly attenuated the induction of both apoptotic markers, whereas only modest changes were observed in HPrEC spheroids. These results provide additional mechanistic evidence that ROS contributes, at least in part, to ATG/CUR-induced apoptotic signaling under 3D tumor-like conditions and extend the apoptotic findings obtained in the 2D model to a more physiologically relevant culture system.

Taken together, the NAC rescue experiments provide functional support for an important role of ROS in linking ATG/CUR-induced redox disruption with metabolic impairment and apoptotic cell death.

Nevertheless, because NAC did not completely restore viability, ATP levels, or apoptotic signaling to control levels in PC-3 spheroids, the present findings do not indicate that ROS is the sole mediator of the anticancer response. Rather, oxidative stress appears to represent a major component of a broader network of metabolic and mitochondrial disturbances induced by ATG/CUR co-treatment.

**Figure 11:**
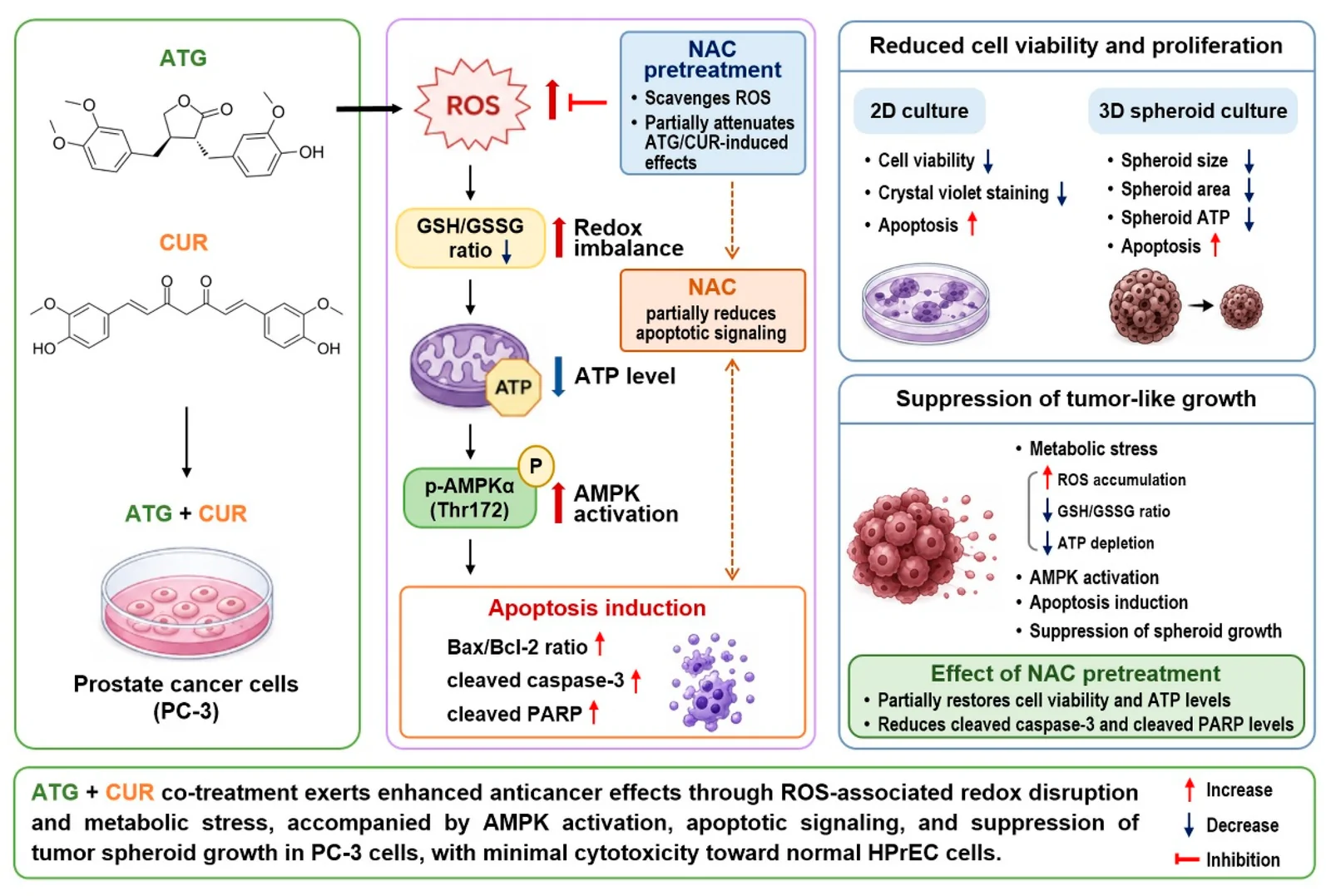
Proposed mechanistic model for the anticancer effects of combined arctigenin (ATG) and curcumin (CUR) treatment in human prostate cancer PC-3 cells. Combined ATG and CUR treatment disrupts intracellular redox homeostasis and cellular energy metabolism, resulting in ROS accumulation, GSH depletion, ATP reduction, AMPK activation, and enhanced mitochondrial apoptotic signaling. These effects are accompanied by an increased Bax/Bcl-2 ratio and enhanced cleavage of caspase-3 and PARP. NAC pretreatment partially restored cell viability and ATP levels, supporting a functional contribution of ROS to the effects of ATG/CUR co-treatment. In addition, NAC pretreatment significantly attenuated ATG/CUR-induced increases in cleaved caspase-3 and cleaved PARP in PC-3 spheroids, providing further evidence for the involvement of ROS in apoptotic signaling under 3D spheroid conditions. These effects were observed in both two-dimensional (2D) monolayer cultures and three-dimensional (3D) spheroid models, with substantially stronger responses in PC-3 than in normal HPrEC cells. Collectively, these findings support an important contribution of ROS-associated redox disruption and metabolic stress to the anticancer effects of ATG/CUR co-treatment; however, the partial rescue by NAC suggests that ROS is unlikely to be the sole mediator of the overall response. Created in BioRender. Lee, Y. J. (2026) https://BioRender.com/3ijwenz.

The mechanistic framework proposed in Figure 11 integrates these molecular events into a unified model encompassing oxidative stress, metabolic dysfunction, AMPK activation, mitochondrial apoptosis, and suppression of prostate cancer cell growth.

The partial rescue observed following NAC pretreatment further supports the involvement of ROS in this model; however, the present data support an association among ROS accumulation, metabolic stress, AMPK activation, and apoptotic signaling rather than establishing an exclusively linear causal pathway.

Taken together, this study identifies coordinated disruption of redox homeostasis and cellular energy metabolism as an important component of the anticancer response to ATG/CUR co-treatment.

### 4.2. Differential Sensitivity of PC-3 and HPrEC Cells to Combined ATG and CUR Treatment

One of the notable findings of the present study was the substantially greater response of PC-3 cells to combined arctigenin (ATG) and curcumin (CUR) treatment compared with normal human prostate epithelial (HPrEC) cells. At the selected concentrations, combined treatment produced a markedly greater reduction in cell viability and proliferation and a stronger apoptotic response in PC-3 cells, whereas the corresponding changes in HPrEC cells were comparatively smaller. Consistent with this differential response, the combination index for the tested ATG/CUR condition was below 1 in PC-3 cells but remained close to 1 in HPrEC cells, indicating a synergistic interaction under the tested condition in PC-3 cells and a near-additive interaction in HPrEC cells.

The differential sensitivity of PC-3 and HPrEC cells may be related, at least in part, to differences in their metabolic and redox characteristics. Compared with normal epithelial cells, malignant cells commonly undergo extensive metabolic reprogramming, exhibit increased energetic demands and elevated basal reactive oxygen species (ROS), and show greater dependence on antioxidant defense systems to maintain redox homeostasis [7,8,9,10,11,12,13,14,15,16,31,32,33,34,35,36,37,38,39,40]. Such characteristics may reduce the capacity of cancer cells to tolerate additional oxidative and metabolic stress. In the present study, combined ATG and CUR treatment produced substantially greater ROS accumulation, reduction in the GSH/GSSG ratio, decreases in ATP levels, and apoptotic responses in PC-3 cells than in HPrEC cells, consistent with this interpretation.

These differential responses were also evident in the 3D spheroid model, with particularly clear cancer-to-normal differences observed for several endpoints. Three-dimensional spheroids develop spatial gradients of oxygen, nutrients, metabolites, and drug penetration and establish more extensive cell–cell and cell–matrix interactions than conventional monolayer cultures [51,52,53,54,60]. These additional constraints may amplify pre-existing differences in stress tolerance between cancer and normal epithelial cells and may therefore contribute to the greater cancer-to-normal differences observed for some endpoints in the present 3D model.

However, this interpretation remains inferential because oxygen and nutrient gradients, metabolic flux, and drug penetration were not directly measured in the spheroids. Therefore, the enhanced differential response observed in 3D should be regarded as a feature of the present in vitro model rather than evidence of confirmed tumor selectivity in vivo.

ATG and CUR individually regulate multiple pathways associated with oxidative stress, mitochondrial function, cellular metabolism, and apoptosis [21,22,23,24,25,26,27,28,29,30]. Their combined administration may therefore impose a broader cellular stress burden than either compound alone. In PC-3 cells, this combined stress was accompanied by greater redox imbalance, lower ATP levels, and enhanced apoptotic responses, whereas the corresponding changes in HPrEC cells were less pronounced.

Taken together, these findings suggest that the greater growth-inhibitory effect of combined ATG and CUR treatment in PC-3 cells may reflect differential vulnerability to oxidative and metabolic stress rather than nonspecific cytotoxicity.

Further studies using additional prostate cancer and normal prostate cell models will be required to determine how broadly this differential response can be generalized.

### 4.3. Redox Imbalance and Metabolic Stress

Cancer cells frequently maintain elevated levels of reactive oxygen species (ROS) as a consequence of oncogenic signaling and metabolic reprogramming while relying heavily on antioxidant defense systems to preserve intracellular redox homeostasis [31,32,33,34,35,36,37,38,39,40]. This balance may render cancer cells particularly susceptible to additional oxidative stress when their antioxidant capacity is exceeded.

In the present study, combined ATG and CUR treatment markedly increased intracellular ROS levels and decreased the GSH/GSSG ratio in PC-3 cells, whereas substantially smaller changes were observed in HPrEC cells.

Importantly, NAC pretreatment significantly attenuated ROS accumulation and partially restored PC-3 cell viability, providing functional evidence that oxidative stress contributes to the growth-inhibitory response induced by combined ATG and CUR treatment.

Alterations in redox status were accompanied by reductions in cellular ATP levels. Redox regulation and cellular energy status are closely interconnected, and disturbances in these processes can influence the ability of cancer cells to adapt to metabolic stress [14,15,16,41,42,43,57,58]. In the present study, combined ATG and CUR treatment reduced ATP levels substantially more in PC-3 cells than in HPrEC cells. However, because cellular ATP content may also be affected by treatment-induced reductions in viable cell number and metabolic activity, the observed decrease should be interpreted as evidence of altered cellular energy status rather than as direct proof of mitochondrial dysfunction or energetic collapse.

Notably, NAC pretreatment partially restored ATP levels in PC-3 cells, further supporting a relationship between oxidative stress and treatment-associated changes in cellular energy status.

Nevertheless, these findings do not establish a strictly linear causal sequence from ROS accumulation to ATP reduction or to subsequent signaling events.

Previous studies have shown that oxidative stress, redox regulation, and metabolic adaptation are closely linked to cancer cell survival and therapeutic responses [31,32,33,34,35,36,37,38,39,40,41,42,43,57,58,59]. The present findings extend this concept by showing that combined ATG and CUR treatment produces coordinated alterations in redox balance and cellular ATP levels in PC-3 cells.

The ability of NAC to attenuate ROS accumulation while partially restoring cell viability and ATP levels strengthens the evidence that oxidative stress contributes functionally to these responses.

Taken together, these findings support a model in which oxidative and metabolic stress represent interconnected components of the cellular response to combined ATG and CUR treatment. However, the precise directionality and molecular hierarchy among ROS generation, changes in cellular energy status, AMPK phosphorylation, and apoptosis remain to be established through additional mechanistic studies.

### 4.4. Association Between AMPK Phosphorylation and Apoptosis-Related Responses

The present findings indicate that combined ATG and CUR treatment is associated with reduced cellular ATP levels and increased AMPKα phosphorylation (Thr172) in PC-3 cells. AMPK is a master regulator of cellular energy homeostasis and metabolic adaptation, functioning as a metabolic checkpoint that continuously monitors intracellular ATP availability. It coordinates adaptive responses to energetic stress by regulating mitochondrial function, glucose metabolism, autophagy, and cell survival [41,42,43,55,56].

Depending on the magnitude, duration, and cellular context of stress, AMPK signaling can participate in adaptive responses that promote survival as well as in stress-associated responses linked to growth inhibition and apoptosis [41,42,43,44,45,46,47,48,49,55,56]. In the present study, increased AMPK phosphorylation in PC-3 cells was accompanied by enhanced apoptosis-related responses, including an increased Bax/Bcl-2 ratio, elevated levels of cleaved caspase-3 and cleaved PARP, and increased caspase-3/7 activity. Similar increases in AMPK phosphorylation and apoptosis-related protein expression were also observed in PC-3 spheroids. These parallel changes are consistent with previous evidence linking AMPK signaling with metabolic stress and apoptotic regulation [44,45,46,47,48,49,55,56].

However, the simultaneous occurrence of increased AMPK phosphorylation and apoptosis-related responses does not establish that AMPK activation is required for, or directly mediates, the apoptotic response observed in the present study.

The NAC rescue experiments further demonstrated that attenuation of oxidative stress partially restored cell viability and ATP levels and reduced caspase-3/7 activity in PC-3 cells. In the 3D spheroid model, NAC pretreatment also significantly attenuated ATG/CUR-induced increases in cleaved caspase-3 and cleaved PARP. These findings provide functional support for the contribution of oxidative stress to the apoptotic response but do not establish whether AMPK phosphorylation lies upstream or downstream of these effects.

Accumulating evidence indicates that AMPK functions not only as an energy sensor but also as an important integrator of mitochondrial homeostasis, redox regulation, and apoptotic signaling during severe metabolic stress.

Consistent with this broader role, the present findings support an association among oxidative stress, changes in cellular energy status, increased AMPK phosphorylation, and enhanced apoptotic responses following combined ATG and CUR treatment [41,42,43,44,45,46,47,48,49,55,56,57,58,59].

Nevertheless, direct pharmacological inhibition or genetic manipulation of AMPK would be required to determine whether AMPK is necessary for the apoptosis observed in this study. Thus, increased AMPK phosphorylation should be interpreted as an important component of the proposed stress-response framework rather than as a confirmed causal molecular link between metabolic stress and mitochondrial apoptosis.

### 4.5. Evaluation in the Three-Dimensional Spheroid Model

Conventional two-dimensional (2D) monolayer cultures are indispensable for mechanistic investigations but do not fully recapitulate the structural and physiological complexity of solid tumors. Three-dimensional (3D) tumor spheroids provide a more complex in vitro culture context by incorporating cell–cell and cell–matrix interactions and by generating spatial gradients of oxygen, nutrients, metabolites, and drug penetration [51,52,53,54,60]. Accordingly, spheroid models can provide greater biological and physiological relevance within an in vitro setting than conventional monolayer cultures, although they do not reproduce the full complexity of the in vivo tumor microenvironment.

In the present study, the differential response to combined ATG and CUR treatment observed in 2D cultures was also evident in the 3D spheroid model. PC-3 spheroids exhibited greater reductions in spheroid size, viability, and ATP levels than HPrEC spheroids, together with increased apoptosis-related protein expression. These findings indicate that the treatment-associated responses observed in monolayer cultures were retained in a more structurally complex in vitro model [53,54,60].

Furthermore, NAC pretreatment partially restored spheroid viability and ATP levels in PC-3 spheroids and significantly attenuated ATG/CUR-induced increases in cleaved caspase-3 and cleaved PARP. These results provide functional support for the contribution of oxidative stress to the treatment response under 3D culture conditions.

Nevertheless, the 3D spheroid model used in this study remains an in vitro system and lacks several important features of the in vivo tumor microenvironment, including stromal components, immune responses, angiogenesis, vascular perfusion, and systemic pharmacokinetics. Therefore, the present 3D findings should not be interpreted as direct translational validation of tumor selectivity or therapeutic efficacy. Rather, they provide additional support for the biological relevance of the observed responses within a more complex in vitro context [51,52,53,54,60].

Taken together, the 3D spheroid data extend the observations obtained in conventional monolayer cultures while also defining the limits of the present experimental model. The retention of differential sensitivity between PC-3 and HPrEC spheroids, together with the partial NAC rescue of metabolic and apoptotic responses, strengthens the overall evidence for ROS-associated metabolic stress as an important contributor to the effects of combined ATG and CUR treatment.

Further validation using additional prostate cancer and normal prostate cell models, as well as in vivo studies, will be required to determine the broader biological relevance, reproducibility, and potential translational significance of combined ATG and CUR treatment.

### 4.6. Study Limitations and Future Perspectives

Several limitations of the present study should be acknowledged. First, the mechanistic investigations were primarily performed using a single prostate cancer cell line, PC-3. Although PC-3 cells represent a well-established model of advanced prostate cancer, validation of the present findings in additional prostate cancer cell lines with distinct androgen-response and molecular characteristics would improve the generalizability of the findings. In particular, studies using androgen-sensitive and additional androgen-independent prostate cancer models would be valuable for determining whether the differential response to combined ATG and CUR treatment extends beyond PC-3 cells. Further confirmation in patient-derived prostate cancer models would also strengthen the biological relevance of the present observations.

Second, although the three-dimensional (3D) tumor spheroid model more closely mimics certain structural features of the tumor microenvironment than conventional monolayer cultures, it cannot fully recapitulate the complexity of in vivo tumors, including stromal interactions, immune regulation, angiogenesis, vascular perfusion, and systemic drug metabolism. Accordingly, the present 3D findings should be interpreted as evidence obtained in a more complex in vitro model rather than as direct translational validation. Future in vivo studies will be required to evaluate the pharmacokinetics, bioavailability, efficacy, and systemic safety of combined ATG and CUR treatment [51,52,53,54,60].

Another limitation of the original study design was the lack of direct functional assessment of the contribution of oxidative stress. To address this limitation, NAC rescue experiments were incorporated and demonstrated that attenuation of oxidative stress partially restored cell viability and ATP levels and reduced caspase-3/7 activity in PC-3 cells. In the 3D spheroid model, NAC also partially restored spheroid viability and ATP levels and significantly attenuated ATG/CUR-induced increases in cleaved caspase-3 and cleaved PARP. These rescue findings strengthen the evidence that oxidative stress contributes functionally to the treatment response.

However, the precise molecular relationship among ROS generation, changes in cellular energy status, AMPK phosphorylation, and apoptosis remains incompletely defined. In particular, because AMPK was not directly inhibited pharmacologically or genetically, the present study cannot establish whether increased AMPK phosphorylation is necessary for the apoptotic response. Future studies using pharmacological inhibition or genetic modulation of AMPK signaling will be required to clarify its causal role [55,56,57,58,59]. In addition, comprehensive metabolic analyses, including Seahorse extracellular flux analysis of mitochondrial respiration and glycolytic function, would provide further insight into the treatment-associated changes in cellular energy metabolism.

Despite these limitations, the present study integrates analyses of redox status, cellular ATP levels, AMPK phosphorylation, apoptosis-related responses, and 3D spheroid growth to characterize the cellular response to combined ATG and CUR treatment.

The NAC rescue experiments further provide functional support for the contribution of oxidative stress to the observed growth-inhibitory and apoptotic effects.

Collectively, these findings support a proposed mechanistic framework in which oxidative and metabolic stress are associated with increased AMPK phosphorylation and enhanced apoptotic responses in PC-3 cells, while avoiding the assumption of a fully established linear causal pathway. Further validation in additional prostate cancer models and in vivo systems will be necessary to determine the broader biological relevance, reproducibility, and potential translational significance of combined ATG and CUR treatment.

## 5. Conclusions

In conclusion, combined treatment with arctigenin (ATG) and curcumin (CUR) produced substantially greater growth-inhibitory and apoptotic responses in human prostate cancer PC-3 cells than in normal human prostate epithelial (HPrEC) cells under the tested experimental conditions. These responses were associated with increased reactive oxygen species (ROS) accumulation, disruption of redox balance, reduced cellular ATP levels, increased AMPK phosphorylation, and enhanced apoptosis-related signaling. Importantly, N-acetyl-L-cysteine (NAC) pretreatment attenuated ROS accumulation, partially restored cell viability and ATP levels, and reduced caspase-3/7 activity in PC-3 cells. In the 3D spheroid model, NAC pretreatment also partially restored spheroid viability and ATP levels and significantly attenuated ATG/CUR-induced increases in cleaved caspase-3 and cleaved PARP. These rescue findings provide functional support for an important contribution of oxidative stress to the treatment response. The differential responses observed in 2D cultures were also evident in the 3D spheroid model; however, these findings should be interpreted within the context of a more complex in vitro system rather than as direct translational validation. Overall, the present findings support a proposed model in which oxidative and metabolic stress are associated with increased AMPK phosphorylation and enhanced apoptotic responses following combined ATG and CUR treatment. Although the NAC rescue experiments strengthen the evidence for a functional role of oxidative stress, the precise causal relationships among ROS generation, changes in cellular energy status, AMPK phosphorylation, and apoptosis remain to be established. Further studies using additional prostate cancer and normal prostate cell models, as well as direct pharmacological or genetic modulation of AMPK and in vivo validation, will be required to determine the broader biological relevance and potential translational significance of these findings.

## Figures and Tables

**Table 1:** Primary antibodies used in the present study.

Target Protein	Supplier	Catalog No.	Host Species	Molecular Weight (kDa)	Working Dilution
Phospho-AMPK (Thr172)	Cell Signaling Technology (Danvers, MA, USA)	2535	Rabbit	62	1:1000
AMPKα	Cell Signaling Technology (Danvers, MA, USA)	2532	Rabbit	62	1:1000
cleaved caspase-3	Cell Signaling Technology (Danvers, MA, USA)	9664	Rabbit	17/19	1:1000
Caspase-3	Cell Signaling Technology (Danvers, MA, USA)	14220	Rabbit	35	1:1000
cleaved PARP	Cell Signaling Technology (Danvers, MA, USA)	5625	Rabbit	89	1:1000
PARP	Cell Signaling Technology (Danvers, MA, USA)	9542	Rabbit	116	1:1000
β-actin	Sigma-Aldrich (St. Louis, MO, USA)	A2228	Mouse	42	1:5000

## Data Availability

The original contributions presented in the study are included in the article; further inquiries can be directed to the corresponding author.
